# Targeting Gα_i2_ in neutrophils protects from myocardial ischemia reperfusion injury

**DOI:** 10.1007/s00395-024-01057-x

**Published:** 2024-05-30

**Authors:** David Köhler, Veronika Leiss, Lukas Beichert, Simon Killinger, Daniela Grothe, Ragini Kushwaha, Agnes Schröter, Anna Roslan, Claudia Eggstein, Jule Focken, Tiago Granja, Vasudharani Devanathan, Birgit Schittek, Robert Lukowski, Bettina Weigelin, Peter Rosenberger, Bernd Nürnberg, Sandra Beer-Hammer

**Affiliations:** 1 Department of Anesthesiology and Intensive Care Medicine, Eberhard Karls University, Tübingen, Germany; 2 Department of Pharmacology, Experimental Therapy and Toxicology, Institute for Experimental and Clinical Pharmacology and Pharmacogenomic, Eberhard Karls University, and Interfaculty Center of Pharmacogenomic and Drug Research, Wilhelmstrasse 56, 72074 Tübingen, Germany; 3 Department of Pharmacology, Toxicology and Clinical Pharmacy, Institute of Pharmacy, University of Tübingen, Tübingen, Germany; 4 Division of Dermatooncology, Department of Dermatology, Eberhard Karls University, Tübingen, Germany; 5 Department of Preclinical Imaging and Radiopharmacy, Multiscale Immunoimaging, Eberhard Karls University, Tübingen, Germany; 6 Cluster of Excellence iFIT (EXC 2180) “Image-Guided and Functionally Instructed Tumor Therapies”, Eberhard Karls University, Tübingen, Germany; 7 Present Address: Department of Biology, Indian Institute of Science Education and Research (IISER) Tirupati, Tirupati, 517507 India

**Keywords:** G-protein-coupled receptors, Cardiac ischemia reperfusion injury, Neutrophil, Endothelial transmigration

## Abstract

Neutrophils are not only involved in immune defense against infection but also contribute to the exacerbation of tissue damage after ischemia and reperfusion. We have previously shown that genetic ablation of regulatory Gα_i_ proteins in mice has both protective and deleterious effects on myocardial ischemia reperfusion injury (mIRI), depending on which isoform is deleted. To deepen and analyze these findings in more detail the contribution of Gα_i2_ proteins in resident cardiac *vs* circulating blood cells for mIRI was first studied in bone marrow chimeras. In fact, the absence of Gα_i2_ in all blood cells reduced the extent of mIRI (22,9% infarct size of area at risk (AAR) *Gnai2*^−/−^ → wt *vs* 44.0% wt → wt; *p* < 0.001) whereas the absence of Gα_i2_ in non-hematopoietic cells increased the infarct damage (66.5% wt → *Gnai2*^−/−^ *vs* 44.0% wt → wt; *p* < 0.001). Previously we have reported the impact of platelet Gα_i2_ for mIRI. Here, we show that infarct size was substantially reduced when Gα_i2_ signaling was either genetically ablated in neutrophils/macrophages using LysM-driven Cre recombinase (AAR: 17.9% *Gnai2*^*fl/fl*^* LysM-Cre*^+*/tg*^* vs* 42.0% *Gnai2*^*fl/fl*^; *p* < 0.01) or selectively blocked with specific antibodies directed against Gα_i2_ (AAR: 19.0% (anti-Gα_i2_) *vs* 49.0% (IgG); *p* < 0.001). In addition, the number of platelet-neutrophil complexes (PNCs) in the infarcted area were reduced in both, genetically modified (PNCs: 18 (*Gnai2*^*fl/fl*^*; LysM-Cre*^+*/tg*^) *vs* 31 (*Gnai2*^*fl/fl*^); *p* < 0.001) and in anti-Gα_i2_ antibody-treated (PNCs: 9 (anti-Gα_i2_) *vs* 33 (IgG); *p* < 0.001) mice. Of note, significant infarct-limiting effects were achieved with a single anti-Gα_i2_ antibody challenge immediately prior to vessel reperfusion without affecting bleeding time, heart rate or cellular distribution of neutrophils. Finally, anti-Gα_i2_ antibody treatment also inhibited transendothelial migration of human neutrophils (25,885 (IgG) *vs* 13,225 (anti-Gα_i2_) neutrophils; *p* < 0.001), collectively suggesting that a therapeutic concept of functional Gα_i2_ inhibition during thrombolysis and reperfusion in patients with myocardial infarction should be further considered.

**Supplementary Information:**

The online version contains supplementary material available at 10.1007/s00395-024-01057-x.

## Introduction

Cardiovascular diseases remain still the leading cause of death worldwide [96, 85]. Among them, 8.9 million people worldwide died from ischemic heart disease in 2019, with acute myocardial infarction (MI), for example, responsible for more than 4 million deaths in Europe and northern Asia, and more than 2.4 million deaths in the US each year [79]. The current standard treatment for MI is based on early myocardial reperfusion through thrombolytic therapy or primary percutaneous coronary intervention [79]. Paradoxically, the restoration of perfusion in the ischemic myocardium causes tissue damage leading to myocardial ischemia reperfusion injury (mIRI) [4, 102]. mIRI manifests from a number of pathomechanisms, including formation of reactive oxygen species (ROS), intracellular calcium overload, edema, vasomotion, microembolization, stasis, intravascular cellular aggregates, and capillary destruction and rupture [14, 31, 32, 45]. So far, efforts to translate effective strategies in animal models to the prevention or treatment of mIRI in patients have been less successful [33].

Neutrophils are among the first cells recruited to sites of tissue damage, where they contribute to tissue repair through the removal of cell debris, to the release of proresolving mediators, and to the discharge of microvesicles but they may also damage remaining healthy tissue [52, 64, 92, 95]. In addition, neutrophils are not only essential for removing pathogens or cell debris but also for the resolution of inflammation promoting return to homeostasis [78]. Numerous studies suggest a detrimental role for neutrophils in mIRI. Activated neutrophils exacerbate tissue damage through the release of ROS, proteolytic enzymes, and pro-inflammatory cytokines, resulting in a positive feedback loop of neutrophil recruitment and activation [10, 92]. In addition, neutrophils might contribute to the no-reflow phenomenon, in which the blockage of capillaries by blood cells impedes the reperfusion of ischemic tissue [10, 32]. Massive migration of neutrophils through the endothelium can lead to loss of tight junctions and consequently to dysfunction of the endothelial barrier [91, 98].

Interestingly, platelet-neutrophil interactions play an important role in a wide range of inflammatory conditions, including bacterial infection and sepsis, pulmonary inflammatory and coronary syndromes [62, 89]. Mechanistically, the formation of platelet neutrophil complexes (PNCs) leads to activation of both cell types triggering the release of cytokines, increased production of ROS, as well as expression of adhesion molecules and cell surface receptors [76]. This activation not only facilitates extravasation of neutrophils and their recruitment to the site of inflammation but also to the site of ischemia, also known as sterile imnflammation or alternatively the ischemic inflammatory response (IIR) [29, 75, 83]. Thus, PNCs may contribute to both the thrombotic and inflammatory processes triggered by mIRI [75, 89], which is also reflected by increased numbers of circulating PNCs in the blood of patients with acute coronary syndrome [58, 65, 74]. Furthermore, the number of PNCs in ischemic myocardial tissue correlated with the extent of IIR and mIRI in animal studies [29, 50].

The numerous heterotrimeric G proteins are subdivided according to the degree of homology of the α-subunits. The G_i_ protein family includes three closely related members, Gα_i1–3_, which are characterized by their sensitivity towards pertussis toxin [42, 71]. The Gα_i1–3_ isoforms share 85–95% of amino acid sequence identity and show overlapping expression patterns. While Gα_i1_ is found primarily in the brain, Gα_i2_ and Gα_i3_ are abundantly expressed in the heart, the endothelium, and the immune system [20]. Many biologic functions of leukocytes, including adhesion and chemotaxis, occur through Gα_i_-mediated signaling [12, 43, 72, 94]. For neutrophilic granulocytes, Gα_i2_ has been shown to be indispensable for their accumulation at different sites of inflammation and for chemokine-induced intravascular arrest [57, 100, 103]. We have previously shown that inhibition of Gα_i2_ signaling in platelets has cardio-protective effects [20] and leads to reduced PNC numbers in the ischemic tissue. However, the functional role of Gα_i2_ specifically in neutrophils remained unclear.

The aim of the present study is to examine the inactivation or inhibition of functional Gα_i2_ signaling in neutrophils/macrophages in a mouse model of mIRI by applying three independent and complementary experimental approaches: (1) investigating bone marrow (BM) chimeras deficient in *Gnai2* only in the hematopoietic compartment, (2) studying mouse mutants deficient in *Gnai2* in neutrophils/macrophages using LysM-driven Cre recombinase, and (3) pharmacologic targeting of Gα_i2_ by i.v.-administered Gα_i2_-specific antibodies.

## Materials and methods

### Mice

Global *Gnai2*-deficient (*Gnai2*^–/–^) mice were backcrossed to a C57BL/6N genetic background for more than ten generations and kept in isolated ventilated cages, and were studied in comparison to wild-type (wt) littermate controls [20]. A specific deletion of *Gnai2* in granulocytes/macrophages (*Gnai2*^*nko*^) was achieved by crossing *Gnai2*^fl/fl^ and *LysM*-Cre^+/tg^ mice [16, 54] and compared to *Gnai2*^fl/fl^; *LysM*-Cre^+/+^ controls (ctrl). These mice were backcrossed for more than ten generations to a C57BL/6N genetic background excluding genes from the initial 129/Ola background. In accordance with the 3R principle, we did not investigate additional controls, such as *Gnai2*^+/+^; *LysM*-Cre^+/tg^ or *Gnai2*^fl/+^; *LysM*-Cre^+/tg^ mice.

C57BL/6N mice receiving antibody treatment (wt^IgG^ or wt^ab^) were obtained from Charles River (Wilmington, MA, USA). Mice of either sex were used at 8–14 weeks of age. All animal experiments were performed according to the ARRIVE guidelines and have been approved by the Animal Care and Use Committee of the local government (Regierungspräsidium Tübingen) and were in accordance with the German animal laws.

### Generation of bone marrow chimeric mice

BM chimeric mice were generated as previously described [50]. Briefly, donor mice (8 to 10 weeks old, 20 to 25 g body weight (b.w.)) were sacrificed, and BM was harvested by flushing tibia and femur. Then cells were centrifuged at 400 × g for 5 min, resuspended and counted. Recipient mice (8 to 10 weeks of age, 20 to 25 g b.w.) were irradiated with a total dose of 10 Gy. After irradiation, the recipients were reconstituted with 1 × 10^7^ BM cells in 150 μl of 0.9% sodium chloride via tail vein injection. In the first 2 weeks after BM transplantation chimera were fed with water containing tetracycline (100 mg/L) and housed in microisolators for 6–8 weeks before experimentation. In total, four groups of BM chimeric mice were generated: (1) BM cells from wt mice were transplanted into irradiated *Gnai2*-deficient mice [wt → *Gnai2*^−/−^] and (2) vice versa [*Gnai2*^−/−^ → wt]. In addition, BM cells were transplanted (3) from wt mice into irradiated wt mice [wt → wt] and (4) from *Gnai2*-deficient mice into irradiated *Gnai2*-deficient mice [*Gnai2*^−/−^ → *Gnai2*^−/−^] as controls (Supplemental Fig. 1A). The efficiency of BM transplantation was verified by immunoblotting (Supplemental Fig. 1B).

### Murine model of myocardial ischemia

The previously described hanging weight system was applied as a well-established and routinely used murine model of mIRI [20, 48, 49]. Briefly, anesthetized mice (pentobarbital, 80 mg/kg b.w.) (Sigma-Aldrich, St. Louis, MO, USA) were placed on a temperature-controlled heating table. Animals were intubated, ventilated and left parasternal thoracotomy was maintained to display the left coronary artery. Coronary artery occlusion was performed for 1 hour and subsequently, the hearts were exposed to a 2-hour reperfusion period. Mice receiving Gα_i2_-specific antibodies (for details of the antibody see Sect. “Immunoblot analysis of Gα_i2_ expression”) were treated with the antibodies 5 min before onset of reperfusion. Afterward blood was collected for detection of troponin I levels and hearts were removed for subsequent analyses. The extent of infarct size was determined according to the state of the art as defined by current guidelines by calculating the percentage of infarction compared with the area at risk (AAR) [13, 59–61]. For this purpose, a double staining technique using triphenyl tetrazolium chloride (TTC) to mark vital and necrotic tissue and Evans Blue staining to negatively mark the AAR was used [27]. Images were taken with an Olympus SZX12 microscope. Planimetric determination of infarct size and AAR was performed using ImageJ Software.

### Electrocardiogram (ECG) records

Mice were anesthetized with pentobarbital-Na^+^ (70 mg/kg *i.p.*) and were positioned on a small animal physiological monitoring system (HPMS, Harvard Apparatus, Holliston, MA, USA) equipped with a tablet (No. 75-1501). Temperature was kept at 37.5 °C throughout the experiment using a rectal probe connected to an integrated heating platform. Paws were placed on gel coated ECG surface electrodes and recordings were performed according to the manufacturer’s instruction manual. Baseline ECG was measured for 5 min, then either anti-Gα_i1/i2_ (1 µg/mouse) or IgG control (1 µg/mouse) antibodies were administered by *i.v.* injection. After 5 min, postinjection ECG responses were monitored continuously for a period of 2 min. The experimenter who evaluated the recordings had no information about whether anti-Gα_i1/i2_ or IgG control antibodies were used. Recorded data were converted to CSV format and signals were displayed in Excel.

### Troponin I measurement

To measure troponin I in the serum of ischemic mice, blood was collected after reperfusion. Serum from chimeric mice was analyzed with the ADVIA Centaur Immunoassay system using the TnI Ultra chemoliuminescent assay (Siemens Healthcare Diagnostics, Fehrenwald, Germany). Serum from mice subjected to other experiments was analyzed using the ELISA kit SEA478Mu (for murine cardiac troponin I Type 3 (TNNI3) as recommended by the manufacturer’s instructions (Cloude-Clone Corp., Houston, TX, USA).

### Detection of PNCs in heart tissue

To detect PNCs within the AAR, hearts were removed after one hour ischemia followed by one hour of reperfusion and processed for further analyses as described previously [20]. Heart sections of 5 µm thickness were embedded in Tissue-Tek (Fisher Scientific, Schwerte, Germany). Immunohistochemical staining was performed with Vectastain ABC Kit (Linaris, Wertheim, Germany). After inhibiting the non-specific binding sites with Avidin blocking solution (Vector, Burlingame, CA, USA), sections were incubated with primary antibodies (rabbit anti-mouse CD41; AbD Serotec, Düsseldorf, Germany) overnight at 4 °C. Tissue sections were then incubated with biotinylated anti-rabbit immunoglobulin for 1 hour followed by Vectastain ABC reagent for 30 min, and then developed using 3,3′-diaminodbenzidine (DAB) substrate (Fisher Scientific, Schwerte, Germany). For neutrophil staining, the procedure was repeated using rat anti-mouse neutrophil antibodies (rat anti-mouse Ly-6-B2/clone 4; AbD Serotec, Düsseldorf, Germany) and histogreen as substrate (Linaris, Wertheim, Germany). Counterstaining was performed using nuclear fast red (Linaris, Wertheim, Germany). Images were taken with the Leitz DM IRB inverted microscope with the Zeiss AxioCamMRC. Of note, the pathologist was blinded to genotype and BM status.

### Measurement of PNC formation in vitro

Mice were anaesthetized with pentobarbital (80 mg/kg b.w.). Blood was drawn from the inferior vena cava and mixed with 3.2% sodium citrate solution (Merck, Darmstadt, Germany) in a 1:1 ratio. Blood samples were stimulated with 20 µM ADP (Sigma-Aldrich, St. Louis, MO, USA) or PBS (Sigma-Aldrich, St. Louis, MO, USA) and incubated at 37 °C for 15 min. Subsequently, the samples were stained with anti-CD45, anti-Ly-6G/C and anti-CD41 antibodies (Biolegend, San Diego, CA, USA) at room temperature for 15 min. Thereafter, erythrocytes were lysed in 2 ml BD FACS lysing solution (BD Bioscience, San Jose, CA, USA). Samples were measured on a BD FACS Canto II and analyzed using FlowJo software (FlowJo, Ashland, OR, USA). PNCs were identified as CD45^+^ Ly-6G/C^+^ CD41^+^ events and expressed as percentage of CD45^+^ Ly-6G/C^+^ neutrophils. In addition, neutrophil numbers from PBS-treated samples were expressed as percentage of CD45^+^ leukocytes and compared between animals to rule out variations in blood composition [29].

### Isolation of human neutrophils

Following approval by the Institutional Review Board of Tübingen University Hospital (ethics committee; 483/2021BO2), human blood samples were taken from healthy donors after written informed consent, and neutrophils were isolated as follows. Whole blood was mixed with dextran solution (2% Dextran, 0.9% NaCl) in a 1:1 ratio and incubated for 30 min until two phases had formed. The upper phase was loaded on Biocoll (1.077 g/ml, Bio&Sell, Feucht, Germany) in a 3:2 ratio and density gradient centrifugation was performed for 30 min at 2000 × g without brake. After removing the supernatant, the pellet containing erythrocytes and granulocytes was resuspended in erythrocyte lysis buffer (C-C-Pro, Vogtei, Germany), incubated for 10 min and subsequently centrifuged for 10 min at 2.000 × g without brake. The remaining pellet containing neutrophils was washed once in PBS and then resuspended in keratinocyte base medium (CELLnTECH, Bern, Switzerland) containing 1.7 mM CaCl_2_. Cell concentration was brought to 3 × 10^6^ cells/ml.

### Isolation of neutrophils from *Gnai2*^nko^ mice

For chemotaxis analysis cells from BM were prepared by flushing tibia and femur of euthanized mice with PBS (Sigma-Aldrich, St. Louis, MO, USA).

For immunoblot analysis, mice were injected intraperitoneally with 300 µl of 4% thioglycollate solution (BD, Franklin Lakes, NJ, USA). Four hours later, the mice were sacrificed, and the peritoneum was flushed with cold PBS (Sigma-Aldrich, St. Louis, MO, USA).

Neutrophils from BM or peritoneal lavage were purified by immunomagnetic separation according to the manufacturer instructions (MACS neutrophil isolation kit, Miltenyi, Bergisch Gladbach, Germany). Purity was confirmed to be above 90% by FACS analysis using CD11b, CD45 and Ly-6G antibodies (BioLegend, San Diego, CA, USA).

### Isolation of macrophages from *Gnai2*^nko^ mice

BM cells were prepared by flushing tibia and femur of euthanized mice with PBS (Sigma-Aldrich, St. Louis, MO, USA). For macrophage maturation, 1 × 10^6^ cells were seeded in 10 cm dishes (bacterial ones) and cultured for one week in the presence of L929 supernatant.

### Immunoblot analysis of Gα_i2_ expression

Isolated neutrophils or macrophages from *Gnai2*^nko^ mice were counted in a hemocytometer, resuspended in SDS lysis buffer (21 mM Tris–HCl, 0.67% SDS, 19 mg/ml 2-mercaptoethanol, 0.4 mM PMSF) at a concentration of 2 × 10^7^ cells/ml and stored at −20 °C. Finally, 2 × 10^5^ neutrophils or macrophages were loaded on 9% SDS polyacrylamide gels containing 6 M urea and blotted onto PVDF membranes (Merck Millipore, Burlington, MA, USA). As controls, lysates from 2 × 10^5^ splenocytes isolated from *Gnai2*^−/−^ and wt mice were loaded.

To verify BM transplantations, heart tissue and 100 µl blood were homogenized in RIPA buffer and protein concentrations were measured by standard BCA method following the manufacturers’ instructions (Thermo Fisher Scientific, Rockford, IL, USA). Protein lysates (30 µg heart and 15 µg blood) were loaded on 12% SDS polyacrylamide gels and blotted onto nitrocellulose membranes as described [47].

Gα_i2_ protein expression was visualized by immunodetection using rabbit polyclonal anti-Gα_i1/i2_ antibodies (1:2000). The anti-Gα_i2_-specific antibodies are directed against the extreme C-terminus of Gα_i2_ which is known to be essential for functional interaction with its coupling GPCR [70]. Similar to the PTX-mediated ADP-ribosylation of a cysteine in the C-terminus of the Gα subunit, this also leads to a functional uncoupling between receptor and G protein. The antibodies were raised against a 21 amino acid comprising C-terminal peptide derived from Gα_i2_, which is identical to Gα_i1_ [11, 55]; Gα_i2_ can be easily distinguished from Gα_i1_ by a different mobility characteristic in the urea-supplemented polyacrylamide gels described above [54, 55, 73, 100], the use of horseradish peroxidase (HRP)-conjugated anti-rabbit-IgG secondary antibodies (1:2000) (Cell Signaling Technology, Danvers, MA, USA) and the Westar Supernova ECL detection system (Cyanagen, Bologna, Italy). Equal loading was verified with rabbit anti-β-actin (1:10,000) (abcam, Cambridge, United Kingdom) or anti-GAPDH (Santa Cruz Biotechnology, Dallas, USA) antibodies. For recombination analysis in *Gnai2*^nko^ mice, Gα_i2_-protein levels were quantified by densitometry using the ImageJ software (NIH, Bethesda, MD, USA) and were normalized to β-actin levels of the same samples.

### Immunoblot analysis of antibody uptake by neutrophils

Isolated human neutrophils were incubated with either anti-Gα_i2_-specific antibodies [54–56, 100] or anti-IgG antibodies (rabbit-IgG isotype control; Invitrogen, Carlsbad, CA, USA) at 37 °C for one hour. To specifically assess cytosolic uptake of the antibodies, cells were washed twice with PBS and then treated with digitonin (50 µg/ml in PBS w/o Ca^2+^ and Mg^2+^) for 5 min at room temperature followed by another 25 min on ice. Afterward, samples were spun down to separate cytosol from membrane and nuclei (membranous fraction). Supernatant (cytosolic fraction) and membranous fraction were mixed with 4 × Laemmli buffer (Carl Roth, Karlsruhe, Germany). Lysates of 4 × 10^6^ neutrophils were loaded on 7% SDS polyacrylamide gels and blotted onto PVDF membranes (Merck Millipore, Burlington, MA, USA). The uptaken antibodies were visualized by immunodetection using HRP-conjugated anti-rabbit IgG (1:100) (Cell Signaling Technology, Danvers, MA, USA) and the Westar Supernova ECL detection system (Cyanagen, Bologna, Italy). To check for purity of cytosol and membranous fraction anti-Hsp90 (Cell Signaling Technology, Danvers, MA, USA, 1:1000) and anti-EEA antibodies (Cell Signaling Technology, Danvers, MA, USA, 1:1000) were used, respectively.

### Immunofluorescent analysis of antibody uptake by neutrophils

Isolated human neutrophils were incubated with either anti-Gα_i2_-specific antibodies [54–56, 100] or IgG antibodies (rabbit-IgG isotype control; Invitrogen, Carlsbad, CA, USA) at 37 °C for one hour. To specifically detect localisation of the antibodies in different cellular compartments, cells were washed twice with PBS and then stained with lysotracker for one hour. Finally, cells were immobilized on glas slides and fixed with 4% formaldehyde.

A Leica Stellaris 8 confocal microscope was used to capture and accurately visualize the antibody-labeled fluorescence-stained neutrophil granulocytes with either anti-IgG or anti-Gα_i2_-antibodies. The images were taken using a 63 × objective. The standard Leica LAS X software (Leica Application Suite X; version 3.7.3.23245) was used for extended zooming, executed Z stacks, Z-stack video animations and 3D representations with Z-stack cross sections.

### Endothelial transmigration assay of neutrophils in vitro

Transendothelial migration assays were performed as described previously [50]. Briefly, the upper chamber of transwell inserts (Fisher Scientific, Schwerte, Germany) covered with Human Microvascular Endothelial Cells-1 (HMEC-1) cell monolayers was filled with 1 × 10^6^ neutrophils. To establish a chemotactic gradient each lower chamber was filled with 1 μM N-formyl-methionyl-leucyl-phenylalanine (fMLP; Merck, Darmstadt, Germany). Neutrophil transmigration studies were performed at 37 °C for 1 hour. Transmigration was assessed by quantification of the enzymatic activity of the azurophilic neutrophil granule protein myeloid peroxidase in the lower chamber. Neutrophils were preincubated with either Gα_i2_- [54–56, 100] or IgG antibodies (rabbit-IgG isotype control; Invitrogen, Carlsbad, CA, USA).

### Chemotactic migration assay

For chemotaxis analysis MACS-isolated neutrophils were resuspended in RPMI and diluted (6 × 10^6^ cells/ml). To maintain their viability, they were kept at 37 °C.

To control and analyze neutrophil migration, µ-Slide *IbiTreat®* chemotaxis chambers (Ibidi GmbH, Gräfelfing, Germany) were used. For murine neutrophil experiments a collagen gel (rat tail collagen 1.6 mg/ml) was prepared with a final cell concentration of 3 × 10^6^ cells/ml. The gel was inserted in the middle chamber and incubated for 30 min at 37 °C to polymerize. Afterward, the lateral chambers were filled with RPMI and fMLP was added to one side with a final concentration of 10 µM right before starting the imaging process. The imaging was performed with the Leica THUNDER Imager Live Cell & 3D Cell Culture System (Leica Microsystems GmbH, Wetzlar, Germany) using a 20× (0.8 NA) objective and brightfield illumination. Multiple image tiles were recorded per condition which covered a total area of 900 × 1800 µm. Time-lapse sequences were obtained for 30 min in total with a frame rate of one image per minute. Neutrophils of *Gnai2*^nko^ and control animals were imaged simultaneously.

To analyze chemotaxis after Gα_i2_ antibody treatment, human neutrophils were preincubated with anti-Gα_i2_-specific antibodies (1:100) or with IgG (1:100) for 30 min, loaded in µ-Slide *Collagen IV* chemotaxis chambers (Ibidi GmbH, Gräfelfing, Germany) and stimulated with 5 µM fMLP.

### Chemotaxis analysis

To analyze neutrophil migration image tiles were stitched using FIJI/ImageJ (NIH, Bethesda, MD, USA) [90] and cells were segmented using WEKA Trainable Segmentation [6]. The segmented cells were filtered based on cell size (< 10 µm^2^, > 50 µm^2^) to exclude cell debris and segmentation artifacts from uneven background. Binary images of segmented neutrophils were then imported to Bitplane Imaris (Version 9.8) for cell tracking. To exclude dead and immobile cells, we filtered the tracks for track length (> 100 µm for murine and > 220 µm for human cells) before plotting migration speed, track length and persistence. The Chemotaxis and Migration Tool 2.0 (Ibidi GmbH) was used to determine the forward migration index towards fMLP (FMI(x)).

### Statistical analysis

To analyze differences between more than two normally distributed groups, a one-way ANOVA with Newman–Keuls or Tukey’s multiple comparison test was performed as indicated in the Figure legends. For differences between more than two not normally distributed groups one-way ANOVA with Kruskal–Wallis test for multiple comparison was performed. To analyze differences between two normally distributed groups, an unpaired Student’s *t* test was used. *P* < 0.05 was considered statistically significant.

## Results

### Hematopoietic *Gnai2*-deficiency reduces cardiac ischemia reperfusion injury

Our previous studies indicated that a global *Gnai2*-deficiency aggravates mIRI [47]. To provide a first insight whether this effect depends on hematopoietic or tissue resident cells, BM transplantations were performed in mice in which the BM was destroyed by lethal irradiation prior to mIRI (for time line of this myocardial ischemia-reperfusion injury model see Suppl. Fig. 1a). Four experimental groups were analyzed in the study as depicted in Suppl. Fig. 1b. Successful reconstitution was confirmed by immunoblot analysis (Suppl. Fig. 1c). Results from control transplantation models (Suppl. Fig. 2, wt → wt and *Gnai2*^−/−^ → *Gnai2*^−/−^) were consistent with those from non-transplanted wt- and *Gnai2*^−/−^-animals [47], confirming that the global absence of Gα_i2_ exacerbates mIRI. *Gnai2*-deficiency in resident tissue cells (wt → *Gnai2*^−/−^) but not in BM cells (*Gnai2*^−/−^ → wt), increased infarct sizes (*n* = 6–7), which are considered the most robust endpoints of cardioprotection studies [34], to an extent observed in mice with global *Gnai2*-deficiency (*Gnai2*^−/−^ → *Gnai2*^−/−^) (Suppl. Fig. 2a–c). In parallel, serum levels of troponin I (*n* = 3–4) were also elevated. In contrast, selective *Gnai2*-deficiency in the hematopoietic compartment (*Gnai2*^−/−^ → wt) protected against mIRI (Suppl. Fig. 2a–c). Although serum levels of troponin I were similar to wt → wt BM chimeras (Suppl. Fig. 2b), infarct sizes were significantly reduced compared to all groups studied (Suppl. Fig. 2a).

### Neutrophil-specific *Gnai2*-deficiency reduces mIRI

**Fig. 1:**
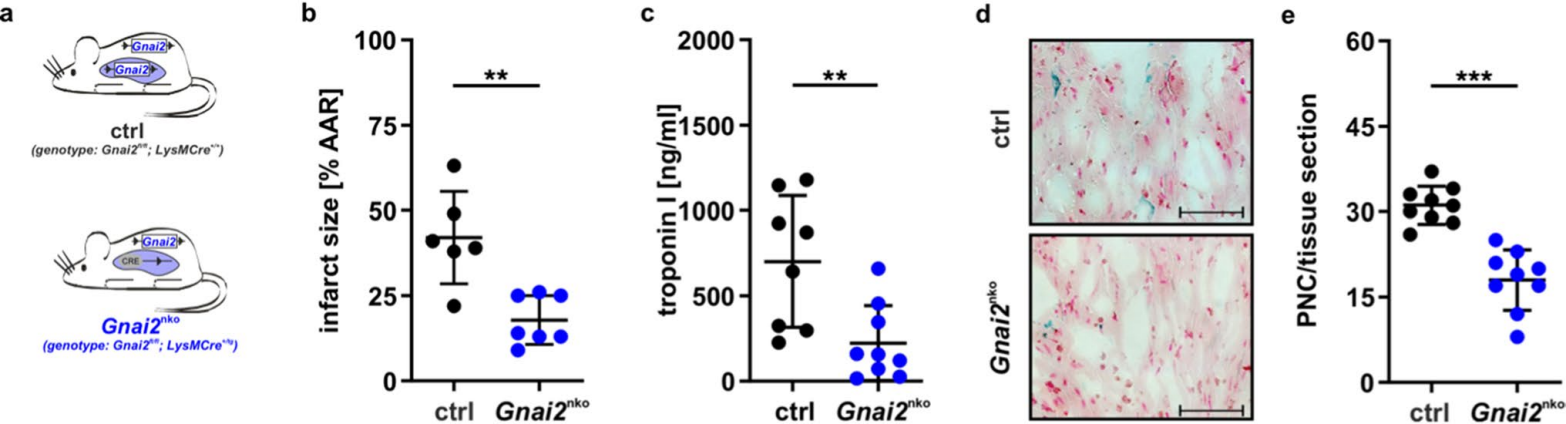
Neutrophil-specific Gnai2-deficiency protects from mIRI. **a** Mouse model to achieve neutrophil-specific *Gnai2*-deficiency (*Gnai2*^nko^). **b** Infarct size determined after 1 h of myocardial ischemia followed by 2 h reperfusion in *Gnai2*^nko^ (blue; *Gnai2*^nko^; genotype: *Gnai2*^fl/fl^; LysM-Cre^+/tg^) mice compared to ctrl (black; ctrl; genotype: *Gnai2*^fl/fl^; LysM-Cre^+/+^). Percentage of necrotic tissue within the AAR is significantly reduced in *Gnai2*^nko^ mice (*n* = 6–7 mice). Representative images are shown in Suppl. Fig. 4a. **c** Corresponding serum troponin I levels of *Gnai2*^nko^ and ctrl animals (*n* = 6–7 mice). **d** Representative platelet-neutrophil complex (PNC) staining of ctrl and *Gnai2*^nko^ heart sections. PNCs were stained for neutrophils (anti-Ly-6B2; blue) and platelets (anti-CD41; black). Scale bar 100 µm **e** Number of PNCs present in histologic sections of *Gnai2*^nko^ hearts (blue) is significantly reduced (*n* = 3 animals; with 3 slices counted per animal). Statistical differences were calculated using Students’ *t* test. Data are shown as mean ± SEM; **p < 0.01; ***p < 0.001 as indicated

The interplay between platelets and neutrophils profoundly determines the outcome of mIRI [49, 50, 62]. The specific absence of Gα_i2_ in platelets extensively reduces mIRI, which is accompanied by decreased PNC counts [20]. To address the role of neutrophil Gα_i2_ in mIRI, LysM-Cre^+/tg^ mice [16] were crossed with mice carrying floxed *Gnai2* alleles [41] to generate a neutrophil/macrophage-specific conditional *Gnai2* knockout (*Gnai2*^nko^) and ctrl, which were subsequently exposed to the mIRI model (Fig. 1 and Suppl. Fig. 3). All genotypes were obtained in the expected Mendelian ratios and were viable and fertile. Although our focus is on neutrophils, as it is well described, that in acute models of mIRI neutrophils are the main drivers of injury, whereas the impact of macrophages is still under investigation at this early time point [5, 8, 51], we confirmed recombination efficiency in both neutrophils and macrophages (Supppl. Fig. 3). Importantly, recombination was detectable only in genomic DNA derived from *Gnai2*^nko^ samples, as indicated by the additional 390 bp band representing the knockout locus (Suppl. Fig. 3a). To additionally verify recombination on the protein level, immunoblot analysis of cell-suspensions from neutrophils and macrophages from these mice showed 66% and 98% reduction in Gα_i2_ protein content, respectively (Suppl. Fig. 3b–e), which is comparable to other studies using neutrophils and macrophages from LysM-Cre mice [1, 40].

Interestingly, *Gnai2*^nko^ mice subjected to mIRI, had on average almost 60% smaller infarct sizes and also greatly reduced serum levels of troponin I compared to their ctrl (Fig. 1b,c and Suppl. Fig. 4a). PNCs are important drivers of inflammation [29, 62] and their accumulation in the ischemic tissue is associated with the extent of IIR [50, 66]. In *Gnai2*^nko^ mice the number of PNCs in the infarcted area was reduced by 43% compared to ctrl (Fig. 1d, e).

To address, whether the lack of Gα_i2_ in neutrophils results in decreased formation of PNCs, whole-blood samples were stimulated with ADP in vitro and then the formation of PNCs was quantified by flow cytometry (Suppl. Fig. 5). ADP stimulation of the blood resulted in similar PNC formation in *Gnai2*^nko^ and control animals (Suppl. Fig. 5a), while the percentage of neutrophils in the blood was similar between both genotypes (Suppl. Fig. 5b).

Taken together, these results suggest that the protection of *Gnai2*^nko^ mice from mIRI is driven by impaired PNC migration, reduced IIR and/or chemotaxis but not due to hampered PNC formation as we have previously found for platelet-derived Gα_i2_ [20].

### Impaired chemotaxis of *Gnai2*-deficient neutrophils

**Fig. 2:**
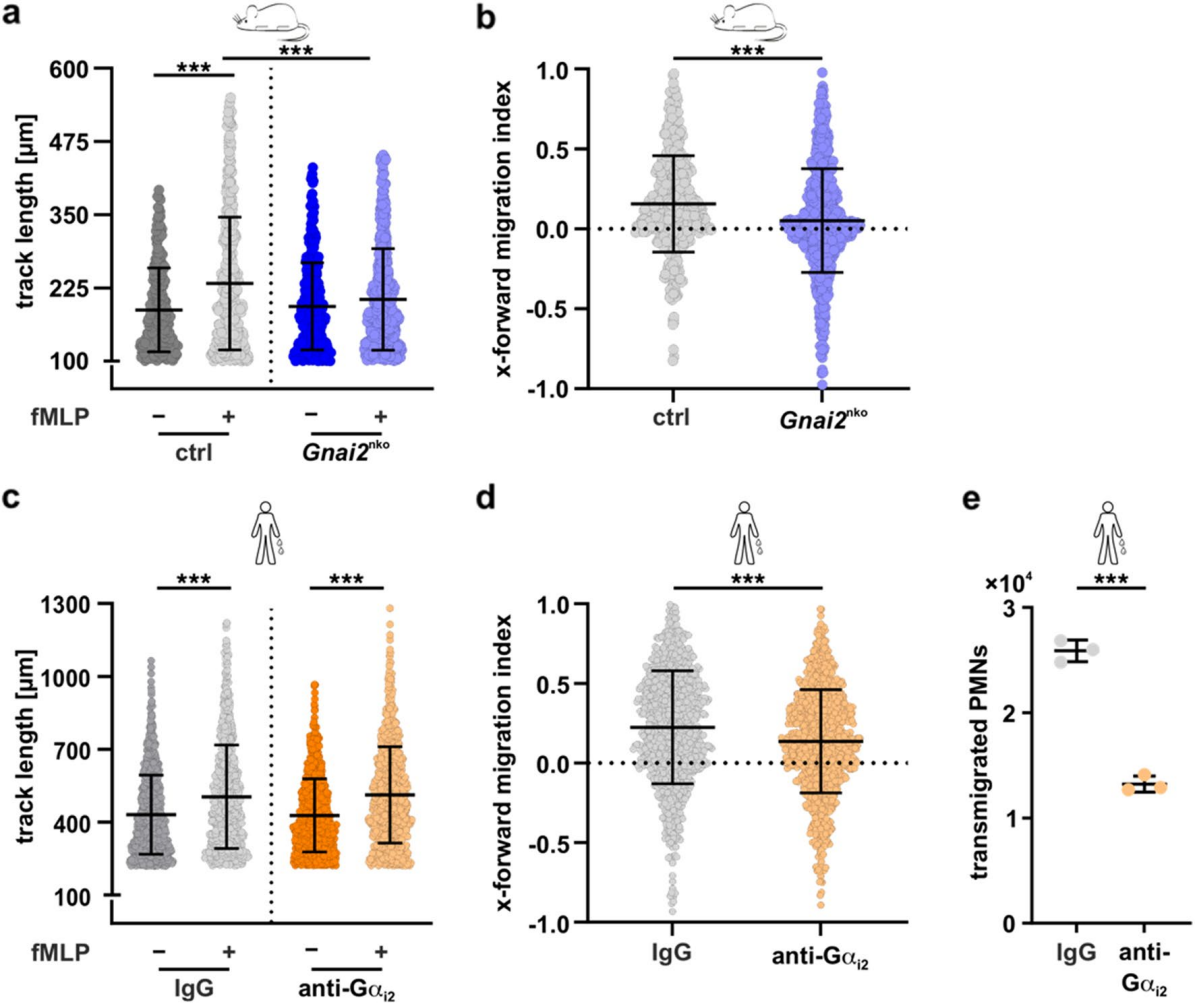
Deletion and inhibition of Gα_i2_ reduce neutrophil migration. **a** Migration of murine neutrophils from control (gray; genotype: *Gnai2*^fl/fl^; LysM-Cre^+/+^) and *Gnai2*^nko^ (blue; genotype: *Gnai2*^fl/fl^; LysM-Cre^+/tg^) mice towards fMLP gradients was monitored by live-cell imaging. The length of fMLP-induced migration tracks (µm) was significantly reduced in *Gnai2*^nko^ (light blue) neutrophils (***p < 0.001, *n* = 282–723 cells per group of four independent experiments). **b** fMLP-induced x-forward migration index (FMI, efficiency of the forward migration of the cells in direction of the x-axis towards the chemotactic gradient) was significantly reduced in *Gnai2*^nko^ neutrophils (***p < 0.001, *n* = 448–723 cells per group of four independent experiments). **c** Directed migration of human neutrophils towards a fMLP-gradient was monitored by live-cell imaging. Human neutrophils were treated with either IgG (gray) or Gα_i2_-specific (orange) antibodies 30 min prior to the assay (***p < 0.001, *n* = 924–1098 cells per group of four independent experiments). **d** The fMLP-induced FMI was significantly reduced in Gα_i2_-specific antibody-treated (orange) neutrophils (***p < 0.001, *n* = 924–1098 cells per group of four independent experiments). **e** fMLP-triggered transmigration of human neutrophils through HMEC-1 monolayer was significantly reduced when treated with Gα_i2_-specific (orange) antibodies. As controls IgG-antibody-treated cells were used (gray). The results are expressed as mean ± SEM (*n* = 3 donors; ***p < 0.001). Statistical differences were calculated using Kruskal–Wallis (**a–d**) or Students’ *t* (**e**) test

To study neutrophil chemotaxis in vitro*,* the directional migration of neutrophils to fMLP, a Gα_i_-dependent chemoattractant, was monitored by live-cell imaging [53, 67, 101]. In control neutrophils, fMLP induced an increase in track lengths (Fig. 2a and Suppl. Fig. 6a) and a stronger orientation of cell tracks towards the chemokine gradient (forward migration index, FMI; Fig. 2b) compared with cells not exposed to a chemotactic stimulus.

In contrast, fMLP-induced track length was significantly shorter in *Gnai2*-deficient neutrophils, which was also reflected in much lower FMI compared with control cells. Thus, the lack of Gα_i2_ in neutrophils impairs movement efficiency and directional migration toward fMLP, suggesting that this results in an impaired PNC migration protecting from mIRI.

### Gα_i2_-specific antibody treatment reduces neutrophil migration and endothelial transmigration

To test whether blocking Gα_i2_ signaling also leads to decreased migration, isolated human neutrophils were treated with Gα_i2_-specific antibodies [54–56] and their migration pattern was monitored (Suppl. Fig. 6b). Although, fMLP-stimulated track length was not affected (Fig. 2c), antibody treatment actually impaired directed migration FMI (Fig. 2d). To rapidly reach the affected tissue after ischemia, neutrophils must first cross the endothelial barrier. Therefore, we also analyzed fMLP-induced transendothelial migration of human neutrophils (Fig. 2e). Interestingly, Gα_i2_-specific antibody treatment also reduced the number of transmigrated neutrophils by 50% compared with control IgG-antibody treatment (Fig. 2e), which could indicate a functionally relevant significance of neutrophil Gα_i2_ for mIRI.

### Gα_i2_-specific antibody treatment protects from mIRI

**Fig. 3:**
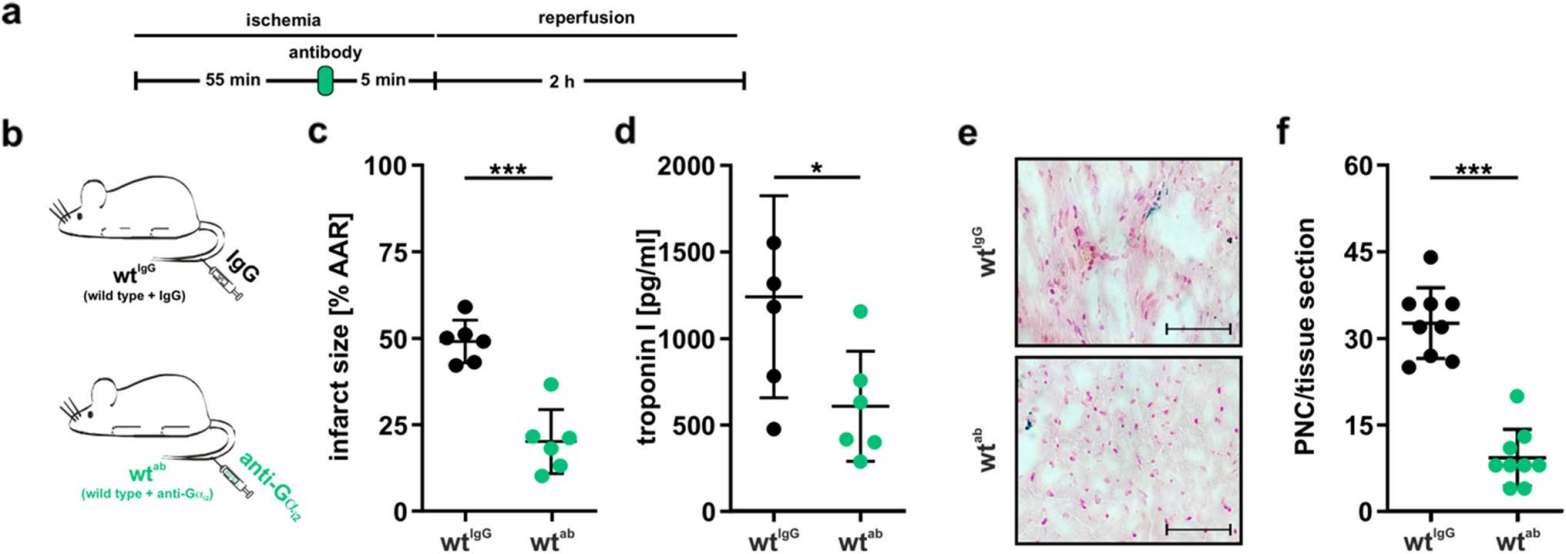
Gα_i2_-specific antibodies protect from mIRI. **a** Timeline for mIRI and antibody treatment. Mice were subjected to 1 h ischemia and then treated with antibodies 5 min before onset of reperfusion. **b** Wild type mice received either IgG (wt^IgG^; black) or Gα_i2_-specific (wt^ab^; green) antibodies (2 µg/mouse) via the tail vein. **c** Infarct size in antibody-treated mice. Percentage of necrotic tissue within the AAR is significantly reduced in wt^ab^ mice (*n* = 6 mice). Representative images are shown in Suppl. Fig. 4b. **d** Correlating serum troponin I levels are significantly reduced in wt^ab^ mice (*n* = 6). **e** Representative platelet-neutrophil complex (PNC) staining of wt^IgG^ and wt^ab^ heart sections. PNCs were stained for neutrophils (anti-Ly-6B2; blue) and platelets (anti-CD41; black). Scale bar 100 µm **f** Number of PNCs present in histologic sections of wt^ab^ hearts (green) is significantly reduced (*n* = 3 animals; with three slices counted per animal). Data are shown as mean ± SEM; *p < 0.05; ***p < 0.001 as indicated. Statistical differences were calculated using Students’ *t* test

**Fig. 4:**
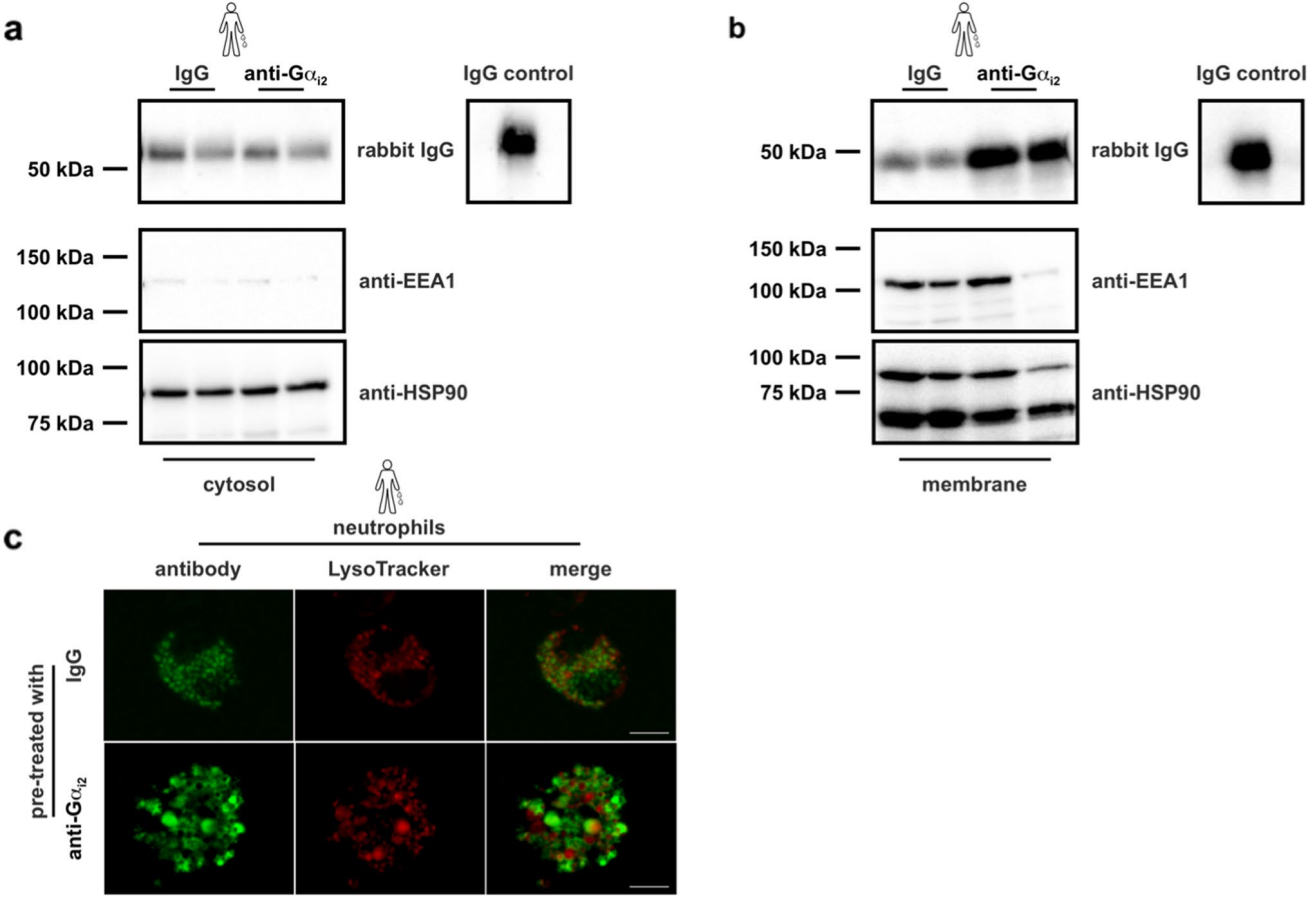
Antibodies are taken up by neutrophils. Antibody visualization in **a** cytosolic and **b** membranous fractions of 1 × 10^6^ human neutrophils by immunoblot analysis. Details of the experimental setup are described in Material and Methods. IgG and Gα_i2_-specific antibodies were detected by using HRP-coupled anti-rabbit-IgG antibodies. As positive control IgG antibodies were loaded and are depicted on the right. EEA1 and HSP90 served as controls for purity of cytosol and membrane fractions, respectively. One representative immunoblot out of three independent experiments is shown. **c** Confocal microscopy of human neutrophils treated with either IgG or Gα_i2_-specific antibodies, detected by using anti-rabbit-IgG antibodies (green). Subsequently phagolysosomes were stained with lysotracker (red). One representative Z-stack image is shown. The reconstructed 3D z-stacks (Suppl. Fig. 10) and movies can be found in the supplemental material

To evaluate the effects of antibody-mediated blockage of neutrophil G_i_ signal transduction on mIRI in our mouse model, wt animals were treated with either control IgG or Gα_i2_-specific antibodies immediately before reperfusion (Fig. 3a, b). Treatment with Gα_i2_-specific antibodies resulted in more than 50% reduction in infarct size compared with IgG-treated animals (Fig. 3c and Suppl. Fig. 4b). Consistent with the protective effect on infarct size, troponin I levels (Fig. 4d) and the numbers of PNCs present in infarct tissue (Fig. 4e, f) were also reduced.

Notably, antibody treatment did not affect heart rate, bleeding time, organ weight or cellular composition in BM, blood and spleen (Suppl. Figs. 7, 8). In conclusion, a single injection of Gα_i2_-specific antibodies prior to reperfusion protects from mIRI.

### Gα_i2_-specific antibodies are detectable intracellularly in neutrophils

As G_i_ proteins are located at the inner leaflet of the plasma membrane Gα_i2_-specific antibodies have to enter the cell to target Gα_i2_. In the cytosol of human neutrophils, we detected IgG in both Gα_i2_-specific and isotype IgG-treated cells (Fig. 4a). The absence of Early Endosome Antigen 1 (EEA1), but the detection of Heat Shock Protein 90 (HSP90) confirmed the purity of the cytosolic fraction. Of note, both antibodies were also detected in the membranous fraction, with higher levels of Gα_i2_-specific antibodies (Fig. 4b). In addition, confocal microscopy revealed different subcellular localization of phagolysosomes and the uptaken antibodies in human neutrophils (Fig. 4c).

Overall, we demonstrated that the antibodies are taken up by neutrophils and that functional inhibition of the intracellular Gα_i2_ protein is thus possible.

## Discussion

Numerous studies document that mIRI is driven in part by inflammation through multiple interacting pathways, with a notable detrimental role of neutrophils, particularly in the initial phase [91, 98]. Neutrophils are activated during acute MI [21] and rapidly infiltrate the ischemic cardiac tissue together with platelets as PNCs [24]. In addition, in vitro findings show that neutrophils directly damage cardiomyocytes [25]. For instance, they are the primary sources of ROS during myocardial ischemia [22] and their depletion resulted in a striking reduction in infarct sizes in mice, dogs and pigs [30, 50, 82]. We have previously shown, that inhibition of Gα_i2_ signaling in platelets has cardio-protective effects [20]. However, the functional role of Gα_i2_ specifically in neutrophils remained unclear.

In the present study three different, independent and complementary experimental approaches were applied to examine its role in an acute mouse model of mIRI by inactivating or inhibiting neutrophil Gα_i2_ signaling. First, we performed mIRI experiments with murine *Gnai2*-deficient BM chimeras, followed by studies in mice displaying a neutrophil/macrophage-specific knockout of *Gnai2*. Moreover, in an experimental interventional therapeutic approach, we treated wt mice with Gα_i2_-specific antibodies. All these approaches showed the same, a massive reduction of cardiac tissue damage. The reduction in infarct size was associated with a reduced chemotaxis and endothelial transmigration of neutrophils in vitro and a reduced presence of PNCs in the infarcted tissue in vivo. These findings strongly suggest a key role of neutrophil Gα_i2_ in the early phase of mIRI, whereas the functional importance of concomitant genetically targeted macrophages during this early stages of mIRI remains to be clarified. Accordingly, we observed that a blockade of Gα_i2_ signaling resulted in an inhibition of human neutrophil functions.

This supports our hypothesis that the loss of function of human neutrophils as a result of inhibition of Gα_i2_ signaling should lead to a reduction in reperfusion injury in the myocardium. Therefore, we envision that neutrophil Gα_i2_ can be a potential target for early pharmacologic treatment of mIRI.

We showed that platelet Gα_i2_ signaling is essential for the activation of neutrophils to form PNCs [20]. Accordingly, ADP-stimulated PNC formation in global *Gnai2*-deficient mice was reduced (Suppl. Fig. 9a) but intact in *Gnai2*^nko^ mice and neutrophils treated with Gα_i2_-specific antibodies (Suppl. Figs. 5 and 9) [20, 29, 50, 62]. Thus, it is unlikely that the reduced infarct sizes after deletion of *Gnai2* in neutrophils can be explained by impaired PNC formation. Rather, our findings suggest a different function of Gα_i2_ in neutrophils for their activation upon binding platelets and/or in neutrophil recruitment that is independent of their interaction with platelets.

Reperfusion injury contributes significantly to loss of myocardial tissue upon infarction. As inflammation is a driver of mIRI, its underlying pathways have been targeted in many experimental studies in search for therapy. Trials have not only included broad anti-inflammatory interventions with glucocorticoids, non-steroid anti-inflammatory drugs (NSAIDs) or immunomodulatory agents but also targeted anti-inflammatory interventions directed against the complement system, cytokines, the TGF-β system, integrins, and selectins [38, 88]. Some of them specifically aimed to prevent neutrophil recruitment to the infarct site by blocking cell adhesion molecules. In particular, blockade of CD18 significantly reduced mIRI in animal studies [88], but failed in clinical trials using antibodies directed against CD18 or CD11/CD18 [9, 26, 87]. Likewise, antibodies directed against components of the complement system were able to reduce mIRI in animal models [77, 97], but were unsuccessful in clinical trials [7]. At present, only the use of low-dose colchicine in long-term management may be considered in the current ESC Guidelines for the management of acute coronary syndromes of the European Society of Cardiology [14]. The advantage of our approach is that, for the first time, it is possible to directly interfere with neutrophil signaling and to block neutrophil migration in acute mIRI instead of interfering with platelet activation. Of course, there are still a number of gaps that need to be addressed to successfully translate our basic animal findings to human patients. These include producing a humanized monoclonal antibody, ensuring the uptake of the antibody restricted to neutrophils and its specific interference with Gα_i2_ signaling. Finally, the antibody has to be tested in humans and must successfully complete clinical trials.

An immediate question regarding the therapeutic use of an anti-Gα_i2_ antibody is whether after systemic administration it can reach its target, as G proteins are physiologically localized inside the cell. Accordingly, the antibodies used must actually enter an intact cell to exert their blocking effect by binding to the G proteins. We used a functional active polyclonal peptide antibody that binds to the extreme C-terminus of Gα_i2_ in a selective and specific manner [55]. The antibody is not only directed to a region that is important for receptor-G protein interaction but also for effector regulation by steric hindrance [2, 37, 72]. Indeed, our data show that it is a functionally active antibody that is effective in vivo. Previously it has been shown that global deficiency of *Gnai2* greatly reduces neutrophil recruitment to sites of inflammation, e.g., in LPS-induced lung-inflammation, LPS-induced peritonitis, skin Arthus reaction and lung Arthus reaction [100, 103]. In this study, targeting intracellularly localized Gα_i2_ by Gα_i2_-specific antibodies disrupted transendothelial migration of neutrophils. In line with these findings, we demonstrated a strong reduction of damage in an acute murine model of mIRI. As a prerequisite for the effect of intravenously injected antibodies on blood-cell Gα_i2_ proteins, the antibodies had to enter the cells without ending up in phagolysosomes. Indeed, the uptake of antibodies into living cells has been described in the pathogenesis of several autoimmune diseases, such as systemic lupus erythematodes (SLE) [3, 69]. However, the mechanisms whereby an antibody may penetrate into the cell are still debated [86, 93]. Possible mechanisms for the entry of antibodies include binding to Fc-receptors and subsequent endocytosis, as well as transport via nucleotide transporters, clathrin-associated vesicles or even free transit [69, 86]. Even if our study does not make a contribution to these details, we can still exclude that Gα_i2_-specific antibodies are found in phagolysosomes causing their rapid degradation. Nevertheless, the exact mechanism of how this antibody is acting on neutrophils or macrophages remains to be clarified in further studies.

Targeting neutrophils for cardioprotection is challenging. Different strategies have been pursued either targeting neutrophil-triggered inflammation, inhibiting neutrophil activity, or reducing the neutrophil accumulation in the infarct area [5, 17–19]. These already included approaches to intervene in the G protein signaling pathway, in these cases in the G_s_ protein-mediated signaling axis, to achieve a cardio-protective effect or reduce the infarct size. Metoprolol, a so-called β_1_-selective adrenoreceptor antagonist, is probably best known [17, 28, 44, 63, 99]. According to the ESC Guidelines for the management of acute coronary syndromes [14], *i.v.* administration of metoprolol should be applied before reperfusion therapy. Metoprolol was found to reduce heart rate, infarct size, and inflammation. However, the results following metoporolol in various preclinical animal studies are controversial [36, 39]. Whereas, on the one hand various neutrophil functions, including neutrophil-platelet interaction, neutrophil migration and reduction of coronary microvascular obstruction were reported to be hampered, others could not confirm the reduction of infarct size reduction and area of no-reflow [17, 46]. Not only a number of possible reasons for these apparent contradictions are discussed, which may be related to the animal model used, genetic variations, the experimental conditions, but also the need for further detailed studies on clinically relevant animal models is emphasized [36, 39, 45, 84]. But the molecular mechanism of action also needs to be clarified in more detail: thus, it is currently assumed that metoprolol exerts its infarct-reducing effects through anti-inflammatory inhibition of neutrophil migration and stunning of coronary microvascular obstruction via non-canonical mechanisms, since classical inhibition of the cAMP signaling pathway is unlikely to affect neutrophil migration [17, 28, 36]. Further support for this assumption is coming from studies targeting phosphodiesterase-4 subtype B (PDE4B) for cardioprotection in acute MI via neutrophils and microcirculation [99]. Despite uncertainty about the mode of action of metoprolol or PDE4B inhibition on neutrophils and other target cells that protect them from mIRI, alternative pathways involving biased signaling are conceivable [72].

## Limitations

Although, we provide strong evidence for a protective effect of Gα_i2_ inhibition on acute mIRI, the underlying molecular mechanisms preventing a worsening or prolonged inflammatory repsonse remain to be further elucidated. In addition, future studies should focus on the impact of neutrophil Gα_i2_-signaling on coronary microvascular obstruction by analyzing the no-reflow phenomenon in more detail. For instance, it is still controversial whether intravascular neutrophil plugging is pivotal or whether neutrophil-mediated damage by ROS, proteolytic enzymes, and lipoxygenase products as well as interactions with the endothelium, platelets, and perhaps with myocytes contribute secondarily to the no-reflow phenomenon [15, 23, 68, 80, 81]. Since we conducted our studies using an acute model of mIRI, the role of neutrophil Gα_i2_ in tissue repair also needs to be clarified, but is beyond the scope of this study. In addition, in this study we did not investigate whether Gα_i2_-signaling in macrophages has an effect on cardiac damage, no-reflow phenomenon and tissue repair [35], but this will be clarified in future studies.

## Conclusion

The present study suggests that cell-specific Gα_i2_ could be a potential new target for pharmacologic (co-)treatment of mIRI. The absence of Gα_i2_ in blood cells as well as the neutrophil/macrophage-specific deletion of *Gnai2* reduced cardiac tissue damage, troponin I levels and PNCs in the infarcted tissue. Furthermore, the specific anti-Gα_i2_ antibody directed against the functional important C-terminus (1) inhibited the effect on (a) the development of mIRI damage, (b) the invasion of PNCs into the affected area and (c) the typical increase in serum troponin I levels in our murine IRI model but also (2) reduced directed chemotaxis of human neutrophils, and (3) massively inhibited transendothelial migration of human neutrophils. We therefore conclude that our antibody is actually capable of exerting a specific effect in both neutrophils and the mIRI model. The identification of its exact mode of action is beyond of the scope of the present study but will be analyzed in future. Importantly, in our mIRI mouse model, a single treatment with Gα_i2_-specific antibodies shortly before reperfusion was highly effective and would perfectly meet the therapeutic demands considering the clinical circumstances of MI patients. Further efforts are clearly needed to test the translational potential of our findings.

## Supplementary Information

Below is the link to the electronic supplementary material.

Supplementary file1 (PDF 2773 KB)

Supplementary file2 (DOCX 22 KB)

Supplementary file3 (MP4 1840 KB)

Supplementary file4 (MP4 2942 KB)

Supplementary file5 (PDF 2566 KB)

## Data Availability

All data will be available on request.

## Abbreviations

AAR: Area at risk
BM: Bone marrow
FMI: Forward migration index
fMLP: *N*-formylmethionyl-leucyl-phenylalanine
GPCR: G protein-coupled receptor
HMEC-1: Human microvascular endothelial cells
IIR: Ischemic inflammatory response
MI: Myocardial infarction
(m)IRI: Myocardial ischemia reperfusion injury
PBS: Phosphate-buffered saline
PNC: Platelet-neutrophil complex
ROS: Reactive oxygen species
TTC: Triphenyl tetrazolium chloride
wt: wild-type
