# Supplementary material for: Targeting Gαi2 in neutrophils protects from myocardial ischemia reperfusion injury
Source: Basic Res Cardiol. 2024 May 30;119(5):717–32. doi: 10.1007/s00395-024-01057-x (PMC11461587; doi:10.1007/s00395-024-01057-x)
Supplement: Supplementary file 1 — Supplementary file1 (PDF 2773 KB) [file 395_2024_1057_MOESM1_ESM.pdf]

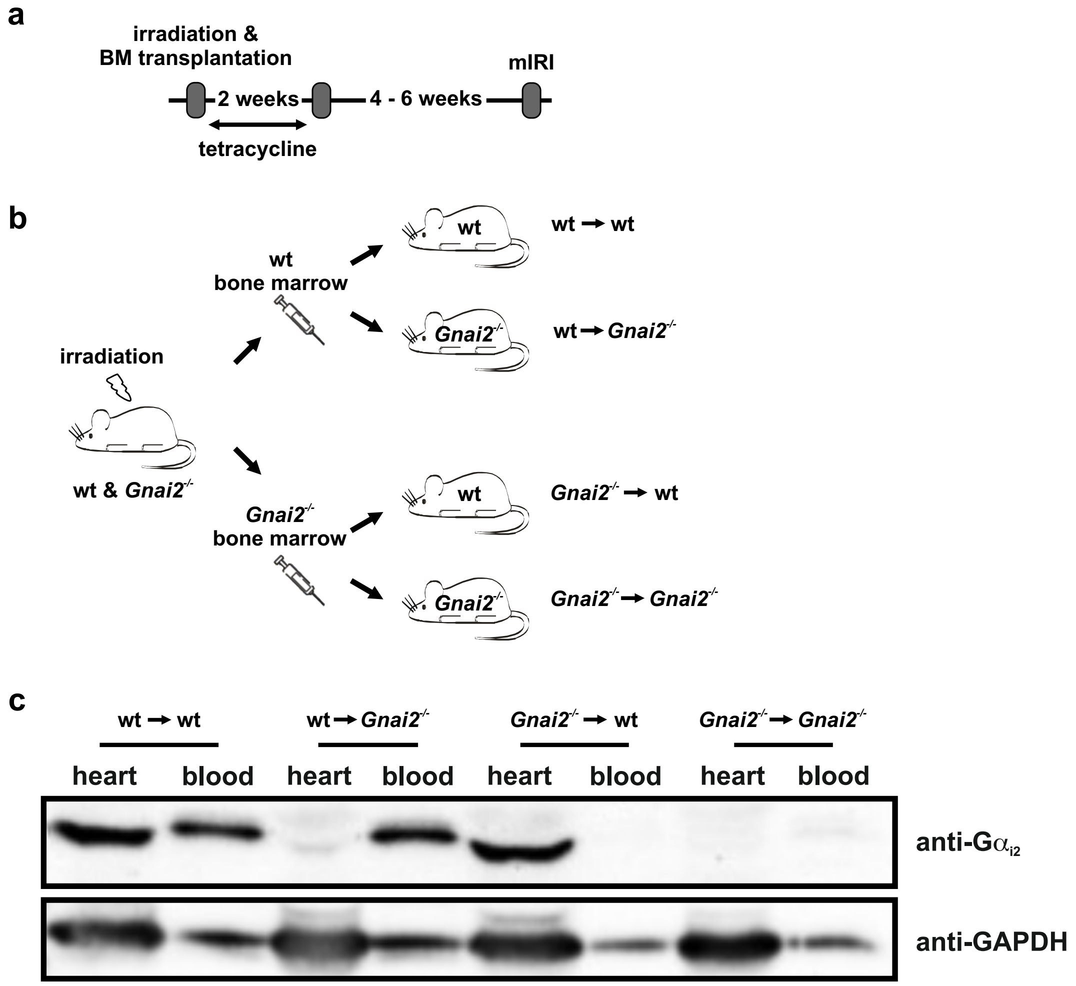

Supplemental Figure 1

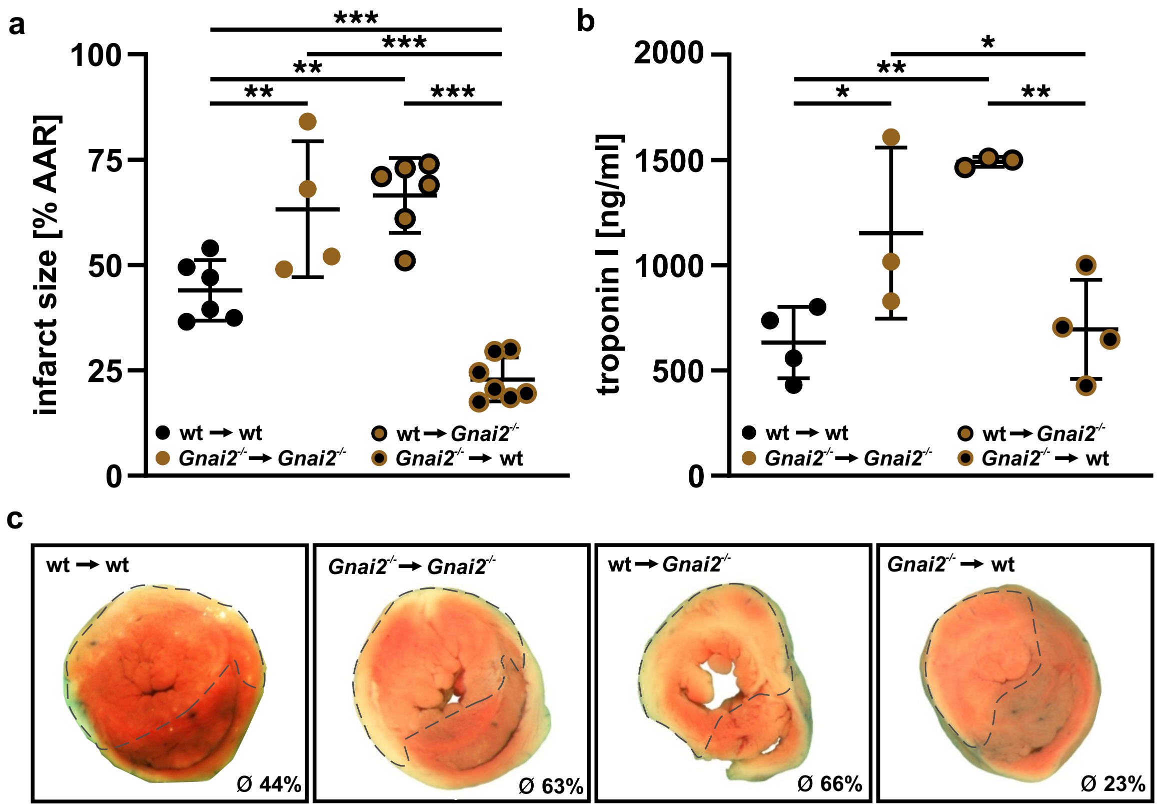

Supplemental Figure 2

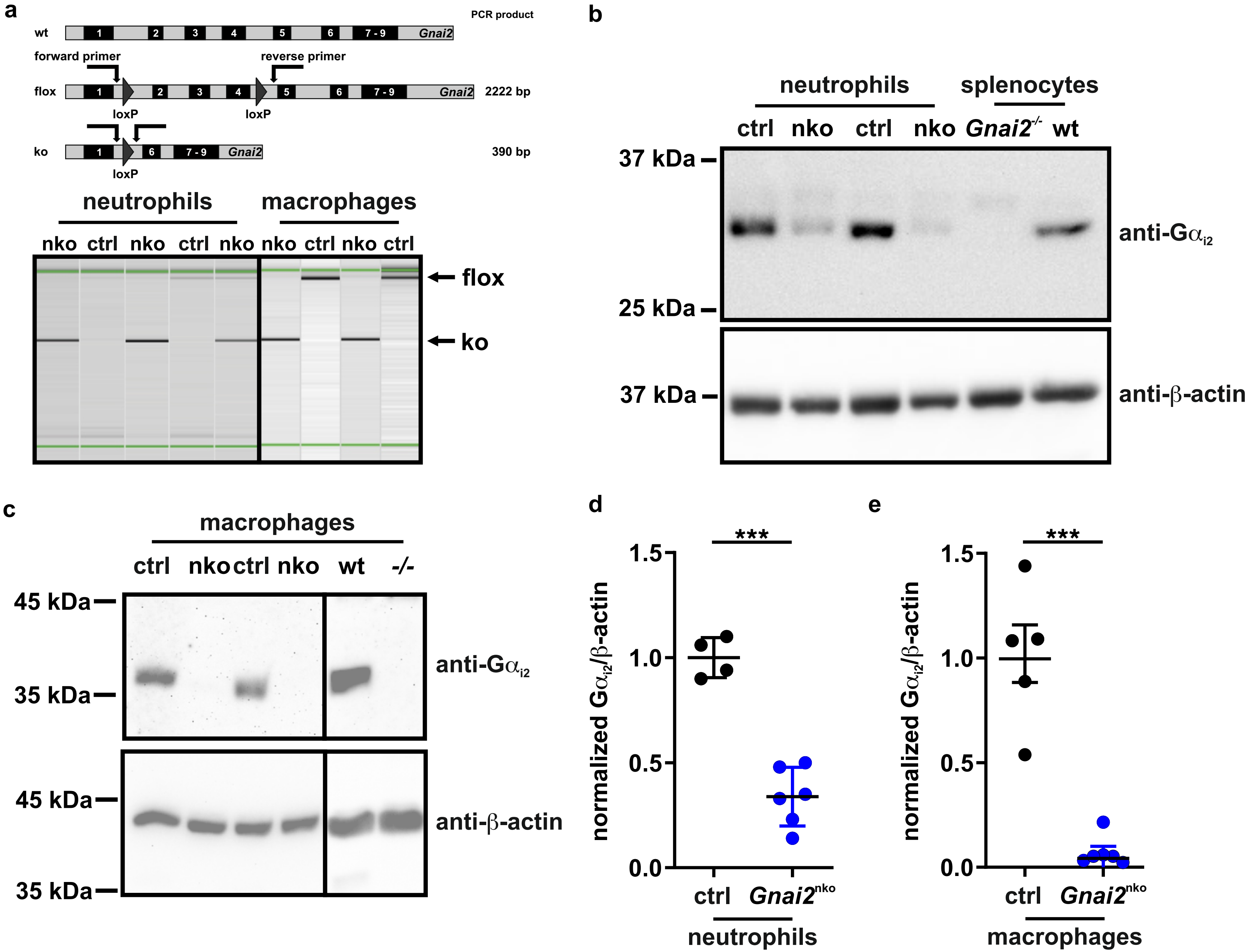

Supplemental Figure 3

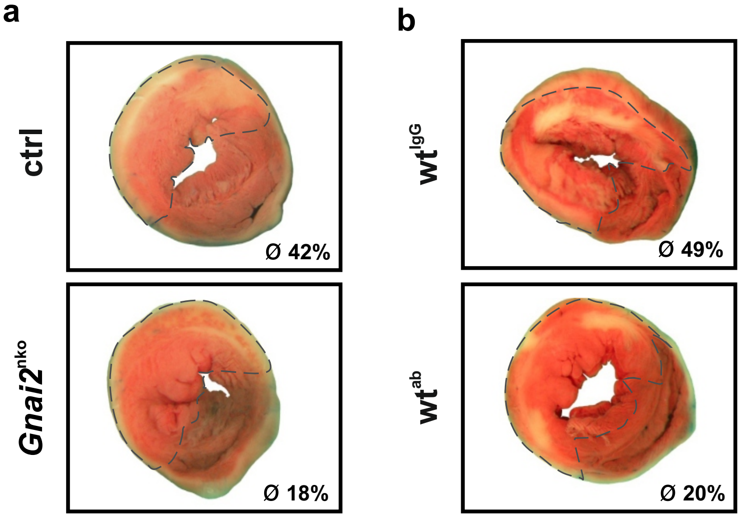

Supplemental Figure 4

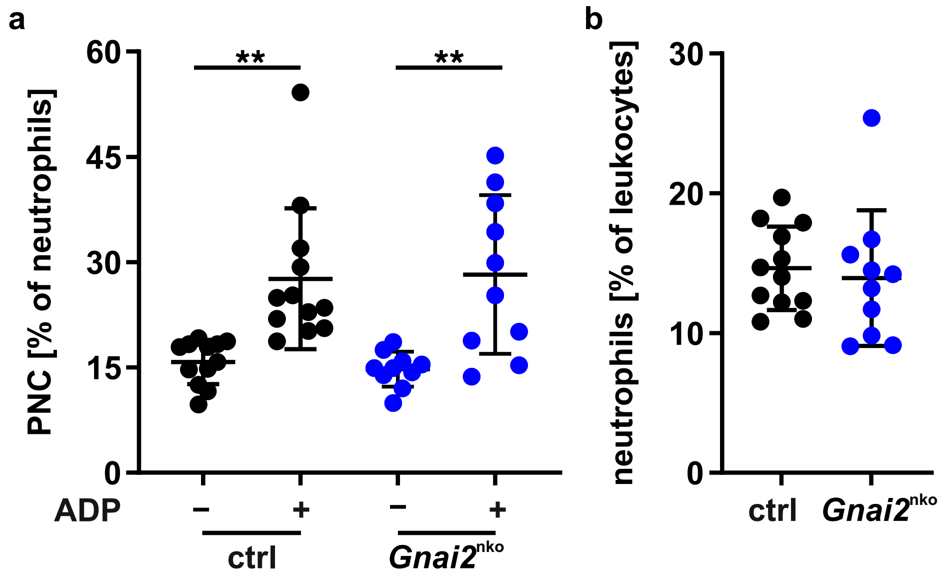

Supplemental Figure 5

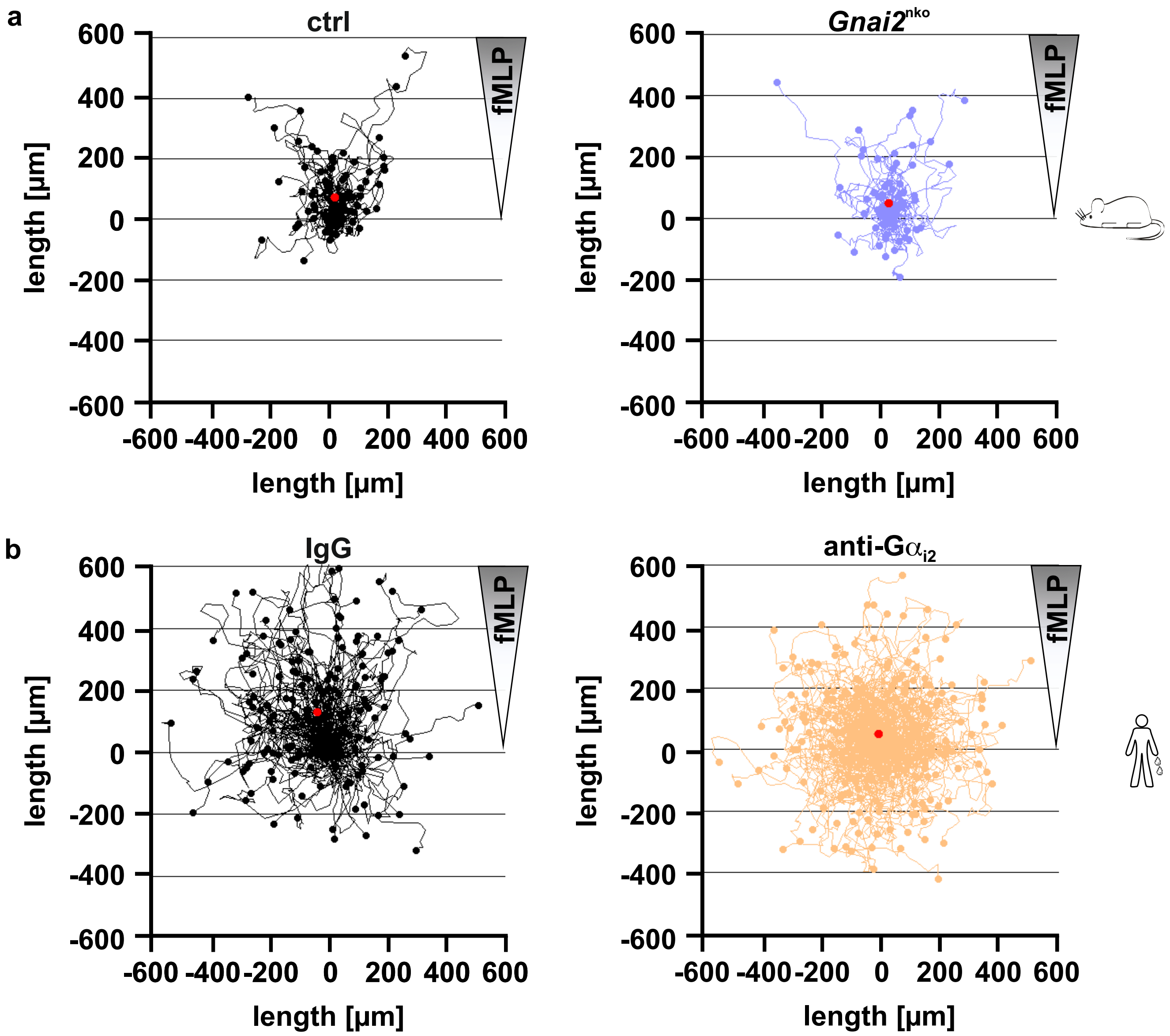

Supplemental Figure 6

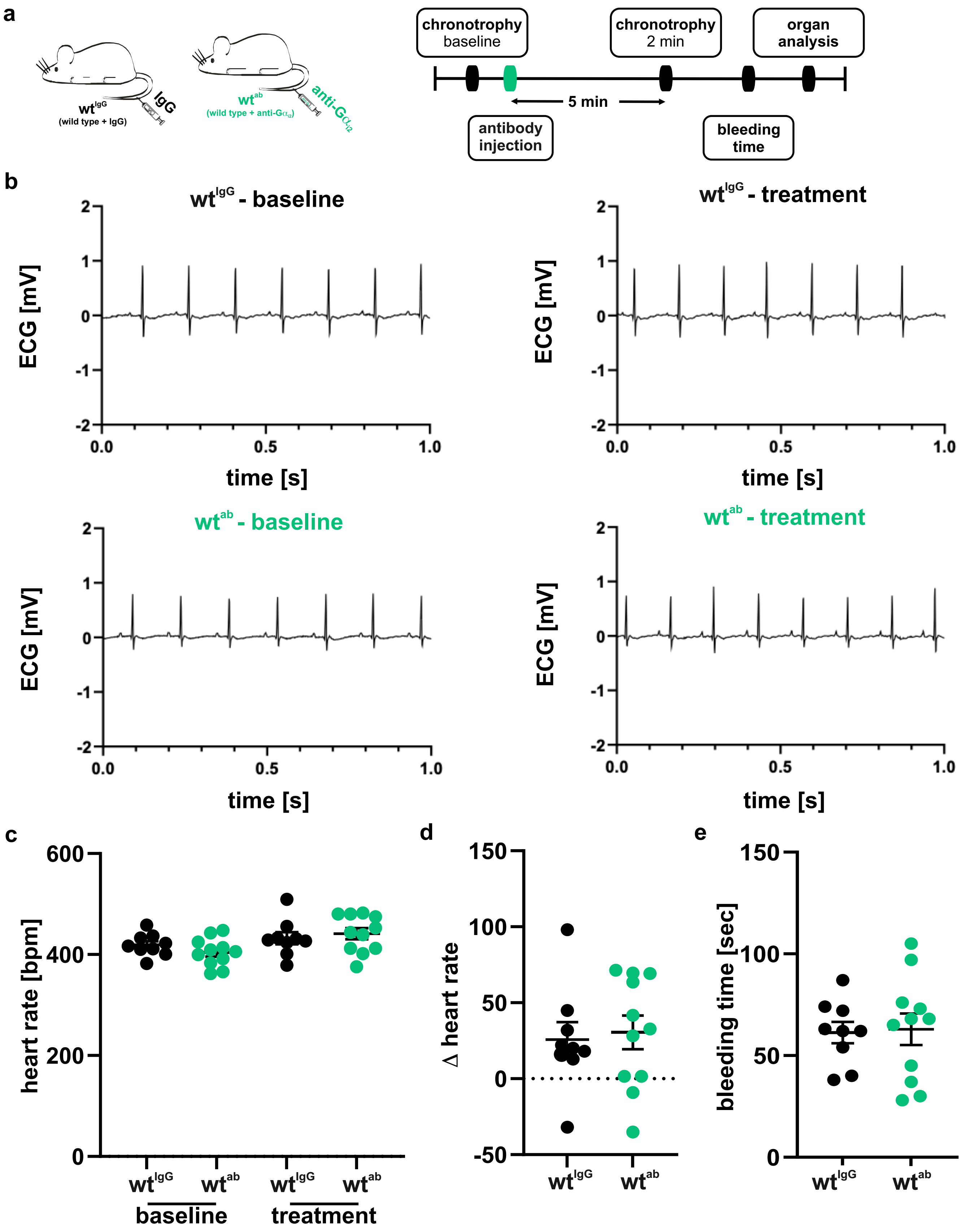

Supplemental Figure 7

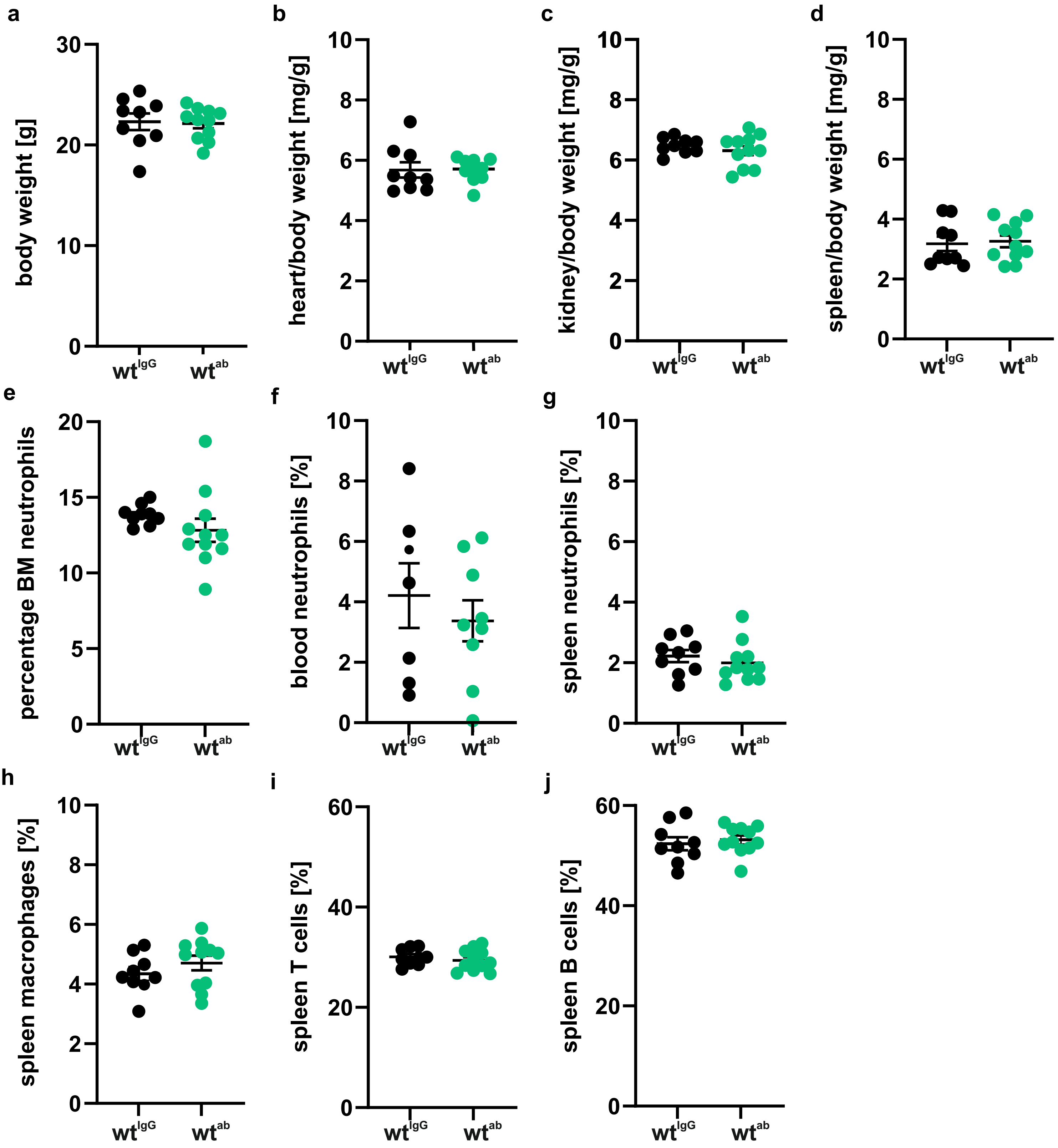

Supplemental Figure 8

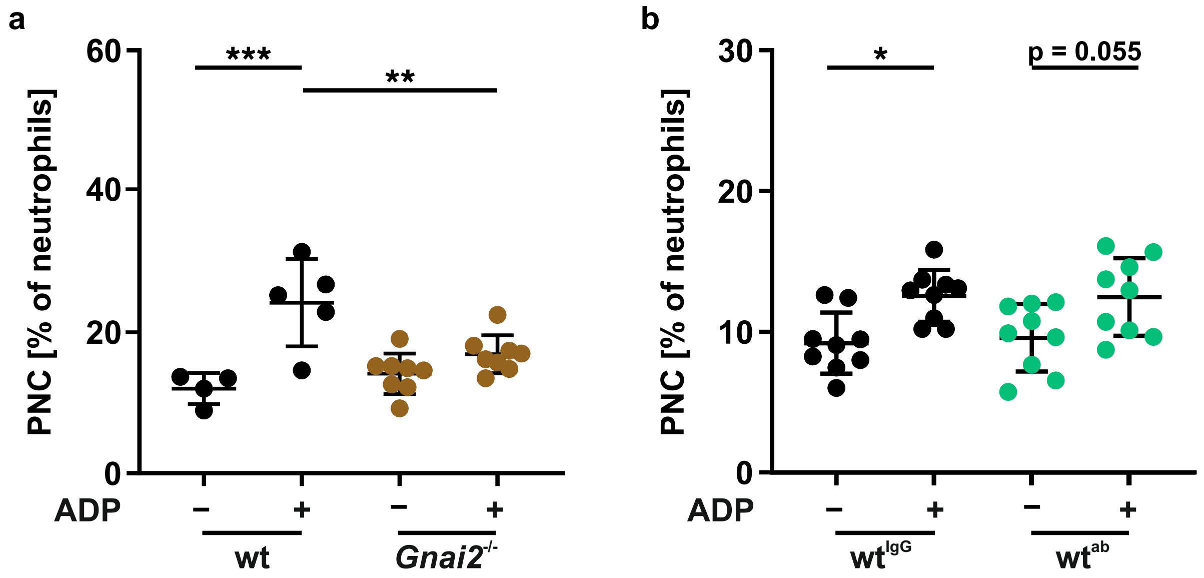

Supplemental Figure 9

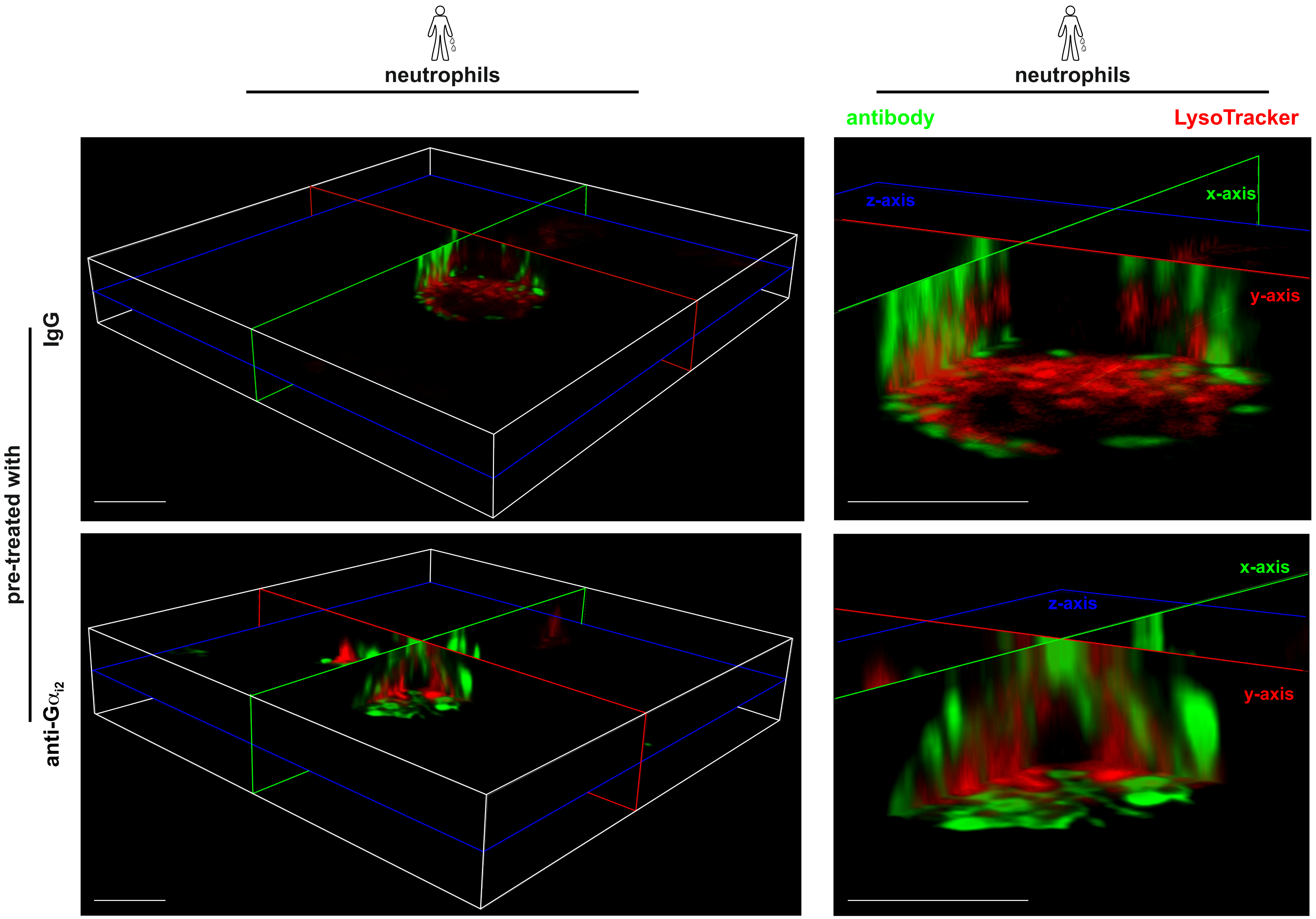

Supplemental Figure 10
