# Supplementary material for: Targeting Gαi2 in neutrophils protects from myocardial ischemia reperfusion injury
Source: Basic Res Cardiol. 2024 May 30;119(5):717–32. doi: 10.1007/s00395-024-01057-x (PMC11461587; doi:10.1007/s00395-024-01057-x)
Supplement: Supplementary file 2 — Supplementary file2 (DOCX 22 KB) [file 395_2024_1057_MOESM2_ESM.docx]

**SUPPLEMENTAL FIGURE LEGENDS**

**Supplemental Figure 1:****Gα_i2_ expression in chimeric animals.**

**(a)** Timeline for the generation of bone marrow (BM) chimeras followed by mIRI. Wt and *Gnai2^−/−^* mice were lethally irradiated at ∼4 weeks of age, transplanted with BM and received tetracycline for two weeks. After four to six weeks animals underwent mIRI. **(b)** Overview of the four groups analyzed: wt mice transplanted with wt BM (wt→wt), wt mice transplanted with Gnai2^−/−^ BM (Gnai2^−/−^→wt), Gnai2^−/−^ animals transplanted with wt BM (wt→Gnai2^−/−^), and Gnai2^−/−^ mice transplanted with Gnai2^−/−^ BM (Gnai2^−/−^→Gnai2^−/−^) **(c)** Immunoblot analysis of hearts and blood from chimeric animals (wt→wt, wt→*Gnai2^−/−^, Gnai2^−/−^*→wt, and *Gnai2^−/−^*→*Gnai2^−/−^*) using Gα_i1/i2_-specific antibodies. Shown is one representative out of two independent immunoblots.

**Supplemental Figure 2: Hematopoietic Gα_i2_ is crucial for myocardial ischemia reperfusion injury (mIRI). (a)** Infarct size after 1 hour of myocardial ischemia followed by 2 hours reperfusion was determined in four groups of bone marrow (BM) transplanted animals: wt→wt (black), Gnai2−/−→Gnai2−/− (brown), wt→Gnai2−/− (brown with black outline) and Gnai2−/−→wt (black with brown outline). Percentage of necrotic tissue within the area at risk (AAR) is significantly reduced in wt mice receiving BM transplants from Gnai2-deficient mice. **(b)** Serum troponin I levels of the corresponding groups are depicted. **(c)** Representative images of heart slices. The AARs are indicated by the dotted lines. Triphenyltetrazolium chloride (TTC) staining marks vital (red) and infarcted (white) tissue within the AAR. A one-way ANOVA with Newman-Keuls multiple comparison was performed to determine statistical significance. Data are shown as mean ± SEM; n = 4-7 mice for AAR and n = 3-4 mice for troponin I; * p < 0.05; ** p < 0.01; *** p < 0.001 as indicated.

**Supplemental Figure 3: Recombination analysis in *Gnai2*^nko^ mice.**

**(a)** PCR strategy and representative genomic DNA analysis to prove Cre recombination in neutrophils and macrophages. Representative genomic DNA analysis showing the 390 bp knockout (ko) and the 2222 bp floxed band in ***Gnai2*^nko^** and only the 2200 bp PCR product in control (ctrl) neutrophils and macrophages. Representative immunoblots of Gα_i2_-expression in **(b)** neutrophils and **(c)** macrophages isolated from ***Gnai2*^nko^** and control mice. Splenocytes from *Gnai2*^–/–^ and wildtype mice served as controls to show antibody specificity. Quantification of relative Gα_i2_ to β‑actin protein levels normalized to ctrl in **(d)** neutrophils and **(e)** macrophages. Data from two (b) and three (c) independent blots with n = 4 ctrl and n = 6 ***Gnai2*^mko^** mice. Data are shown as mean ± SEM; statistics were calculated with Students’ t-test.

**Supplemental Figure 4: Representative images of heart slices.**

**(a)** Representative images of heart slices from ctrl and Gnai2*^nko^* animals. **(b)** Representative images of heart slices from ctrl and wt^ab^ mice animals. The dotted line indicates the AAR. Retrograde Evans blue staining = blue/dark; AAR = red and white; infarcted tissue = white.

**Supplemental Figure 5: Unaltered *in vitro* formation of PNCs in** ***Gnai2*^nko^ mice.**

Whole blood from ctrl (black) or ***Gnai2*^nko^** (blue) animals (n = 10‑12) was treated with PBS or ADP and incubated for 15 minutes at 37°C. Samples were stained with anti‑CD45, anti‑Ly‑6G/C and anti‑CD41 antibodies and analysed by flow cytometry. **(a)** Platelet-neutrophil complexes (PNCs) were identified as CD45^+^ Ly-6G/C^+^ CD41^+^ events and expressed as percentage of CD45^+^ Ly-6G/C^+^ neutrophils. **(b)** Relative neutrophil counts were obtained from PBS-treated samples and are shown as percentage of total leukocytes, which were defined as CD45^+^. Data in (a) and (b) are shown as mean ± SD; statistics were calculated with one-way ANOVA with Tukey’s *post-hoc* test (a) or Students’ t-test (b); ** p > 0,01

**Supplemental Figure 6: Recording of track length of neutrophils.**

All cells of one experiment were graphically recorded within a coordinate system using Chemotaxis and Migration Tool 2.0. The migration starts at 0/0. The fMLP-gradient is depicted in a concentration manner. The red dot indicates the Center of Mass of all moving particles. Representative blots of **(a)** neutrophils from control (left) and *Gnai2*^nko^ (right) mice and **(b)** human neutrophils treated with IgG (left) or Gα_i1/i2_-specific (right) antibodies.

**Supplemental Figure 7: Antibody treatment does not impair heart function *in vivo***

**(a)** Timeline for antibody treatment, heart rate measurements, bleeding time and organ analysis. **(b)** Representative ECG of wt^IgG^ and wt^ab^ mice before (baseline) and after treatment. **(c)** Heart rate measured before (baseline) and 5 min after i.v. injection of either IgG (black) or Gα_i1/i2_-specific antibodies (green). **(d)** Delta heart rate of wt^IgG^ and wt^ab^ mice after treatment. **(e)** Bleeding time of wt^IgG^ and wt^ab^ mice after antibody treatment. Data are shown as mean ± SEM; statistics were calculated with one-way ANOVA with Tukey’s *post-hoc* test (c) or with Students’ t-test (d, e).

**Supplemental Figure 8: Antibody treatment does not affect organ weight or cell composition in lymphoid organs**

**(a)** Body weight of mice treated with either IgG (wt^IgG^; black) or Gα_i1/i2_-specific (wt^ab^; green) antibodies (2 µg/mouse) *via* the tail vein (n = 9 - 11). Organ to body weight ratio of **(b)** heart, **(c)** kidney, and **(d)** spleen from wt^IgG^ and wt^ab^ mice. Percentages of **(e)** BM and **(f)** blood neutrophils of wt^IgG^ and wt^ab^ mice. Percentages of **(g)** neutrophils, **(h)** macrophages, **(i)** T- and **(j)** B-cells in the spleen of wt^IgG^ and wt^ab^ mice. Data are shown as mean ± SEM; statistics were calculated with Students’ t-test.

**Supplemental Figure 9: Antibody treatment does not impair PNC formation *in vitro***

**(a)** Whole blood from wt (black) or Gnai2^-/-^ (brown) mice was treated with PBS or ADP and incubated for 15 minutes at 37°C. **(b)** **wt mice received** either IgG (wt^IgG^; black) or Gα_i1/i2_-specific (wt^ab^; green) antibodies (2 µg/mouse) *via* the tail vein (n = 9). Whole blood was treated with PBS or ADP and incubated for 15 minutes at 37°C. **(a, b)** Samples were stained with anti‑CD45, anti‑Ly‑6G/C and anti‑CD41 antibodies and analysed by flow cytometry. Platelet-neutrophil complexes (PNCs) were identified as CD45^+^ Ly-6G/C^+^ CD41^+^ events and expressed as percentage of CD45^+^ Ly-6G/C^+^ neutrophils. Data in (a) and (b) are shown as mean ± SD; statistical differences were calculated using one-way ANOVA with Tukey’s *post-hoc* test; * p > 0,05).

**Supplemental Figure 10: Uptaken antibodies do not colocalize with LysoTracker**

For the 3D reconstruction, representative cells were placed in a 3D frame and each dimension (x-axis - green; y-axis - red; z-axis - blue) was mapped using the Leica LAS X software (left panels). Magnification of the reconstructed 3D confocal images (right panels) show the distribution of the phagolysosomes stained with LysoTracker (red) and either control IgG antibodies (green, upper panel) or anti-Gα_i2_-antibodies (green, lower panel). Under both conditions no overlay (yellow) of antibodies and phagolysosomes is visible. Scale bars 5 µm. One of the corresponding Z-stack images is shown in Figure 4c. Additionally, Z-stacks are visualized in Suppl. movie 1 (IgG) and movie 2 (anti-Gα_i2_).
