# Supplementary figures and images for: Targeting Gαi2 in neutrophils protects from myocardial ischemia reperfusion injury
Source: Basic Res Cardiol. 2024 May 30;119(5):717–32. doi: 10.1007/s00395-024-01057-x (PMC11461587; doi:10.1007/s00395-024-01057-x)

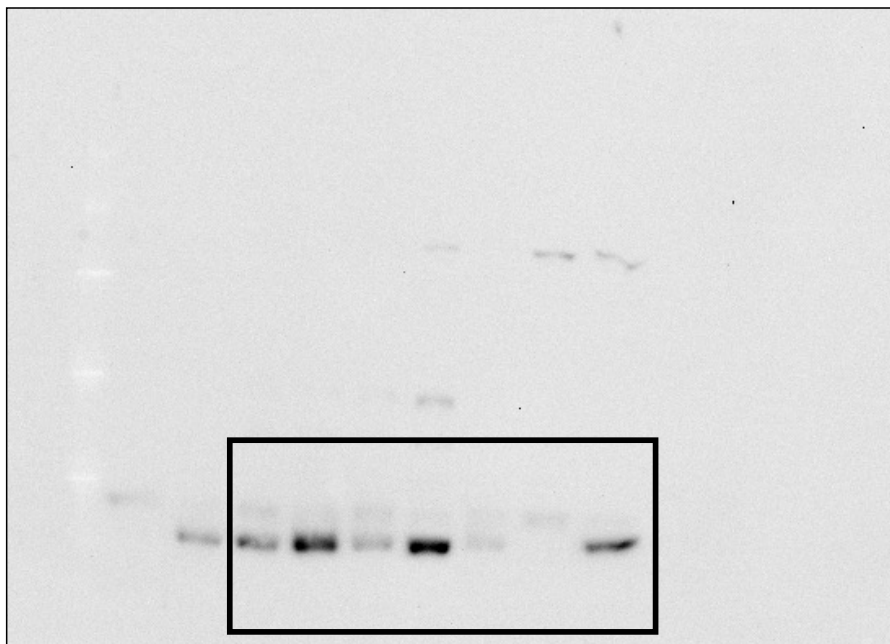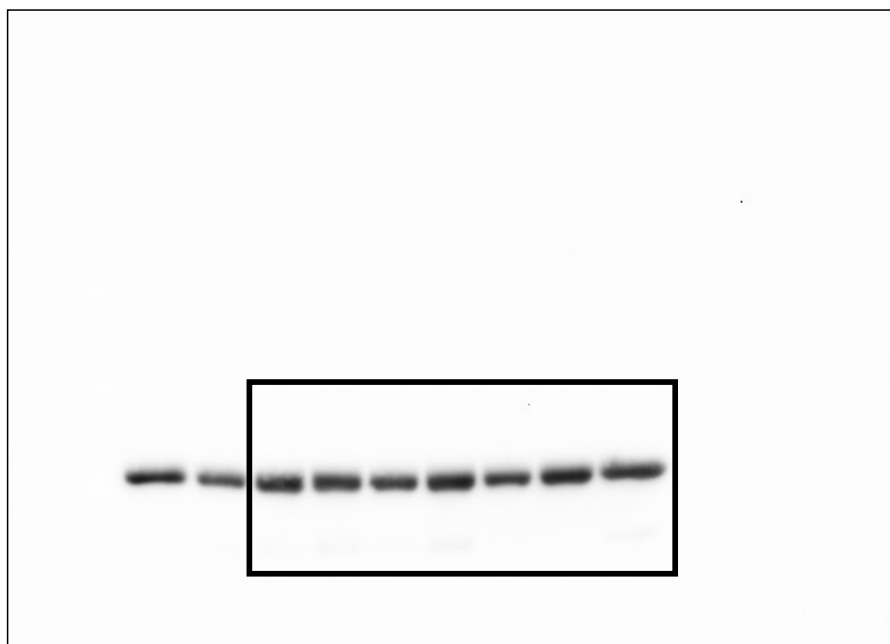

full blots Supplemental Figure 2

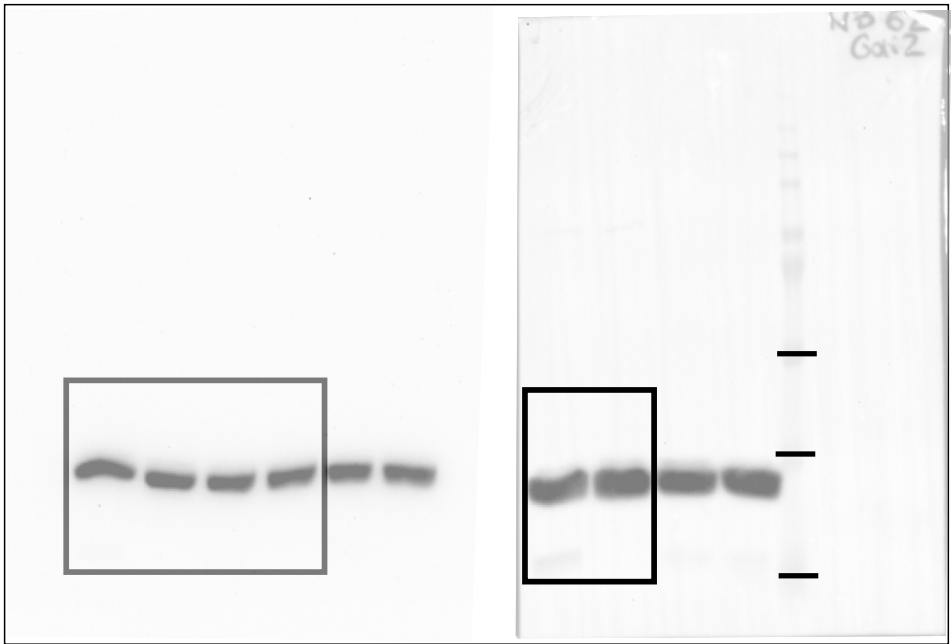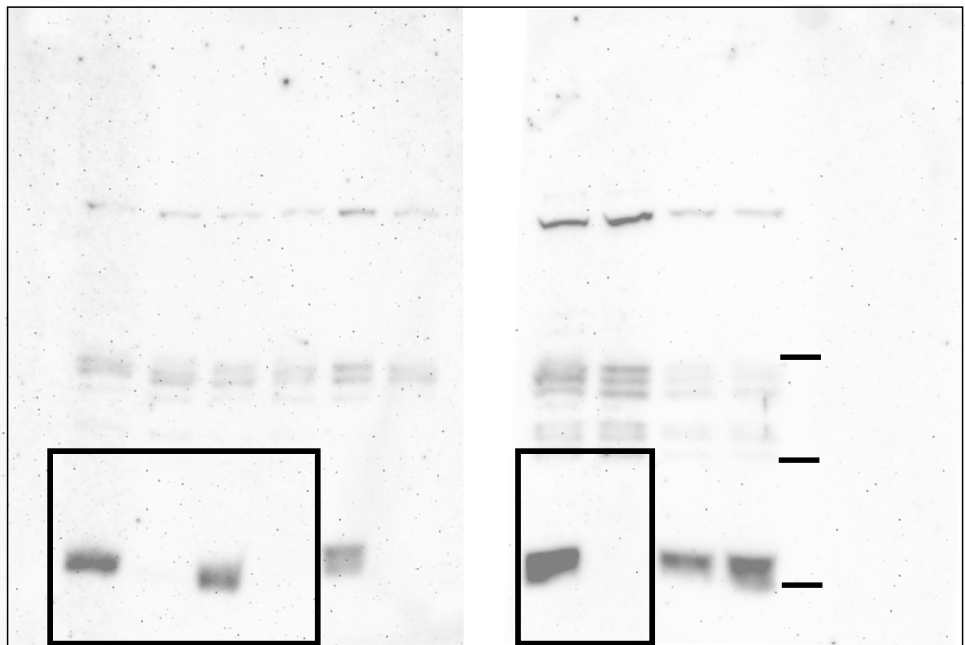

full blots Supplemental Figure 2

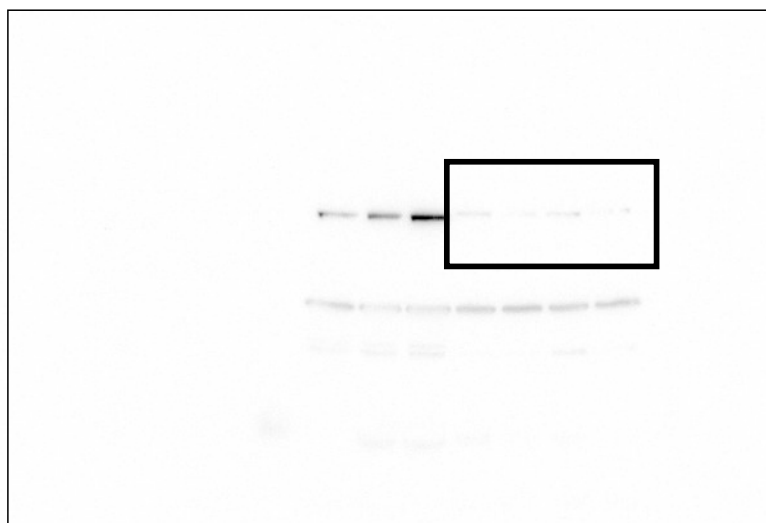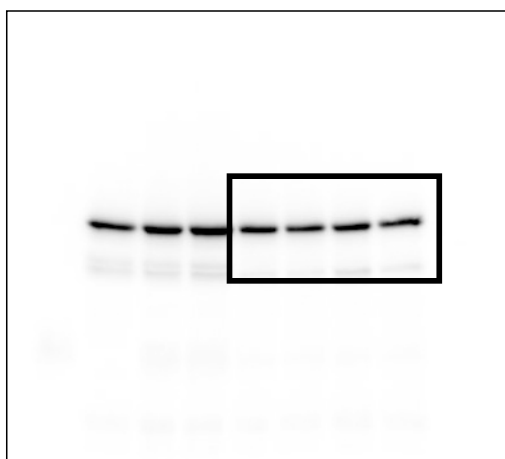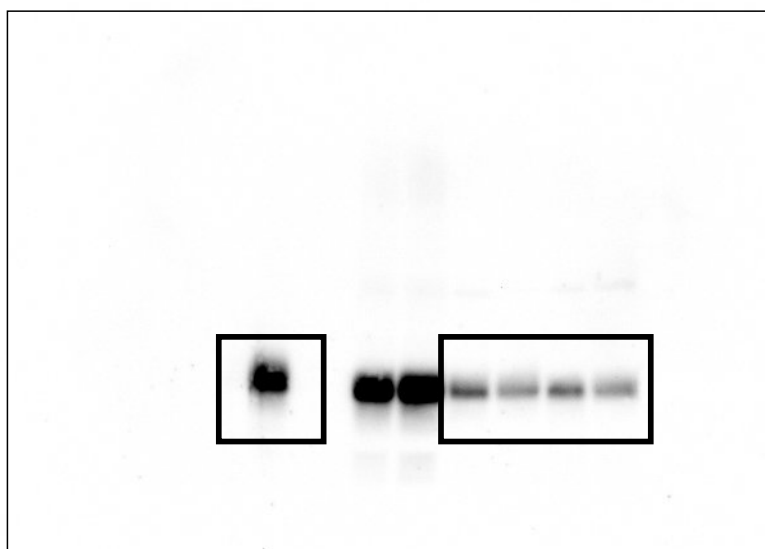

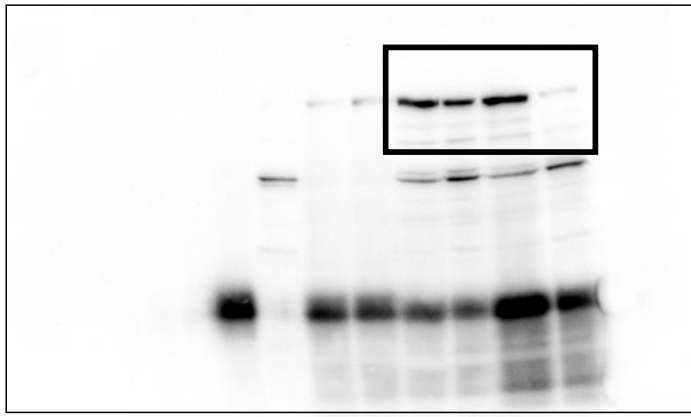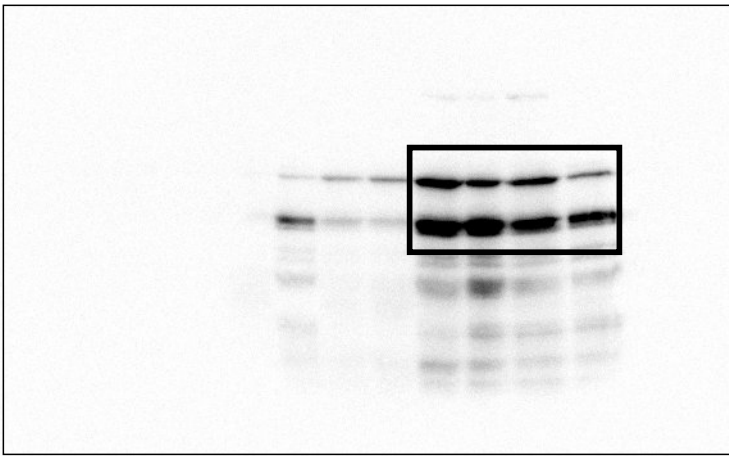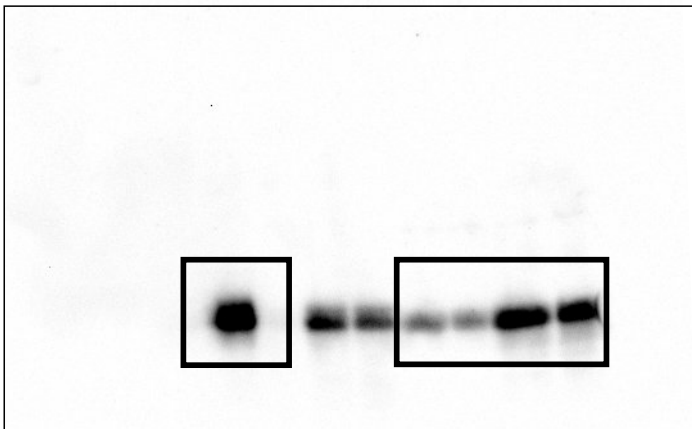

Supplement: Supplementary file 5 — Supplementary file5 (PDF 2566 KB) [file 395_2024_1057_MOESM5_ESM.pdf]
